# Acute depletion of METTL3 implicates *N*^6^-methyladenosine in alternative intron/exon inclusion in the nascent transcriptome

**DOI:** 10.1101/gr.271635.120

**Published:** 2021-08

**Authors:** Guifeng Wei, Mafalda Almeida, Greta Pintacuda, Heather Coker, Joseph S. Bowness, Jernej Ule, Neil Brockdorff

**Affiliations:** 1 Developmental Epigenetics, Department of Biochemistry, University of Oxford, Oxford OX1 3QU, United Kingdom;; 2 The Francis Crick Institute, London NW1 1AT, United Kingdom;; 3 Department of Neuromuscular Diseases, UCL Queen Square Institute of Neurology, Queen Square, London WC1N 3BG, United Kingdom

## Abstract

RNA *N*^6^-methyladenosine (m^6^A) modification plays important roles in multiple aspects of RNA regulation. m^6^A is installed cotranscriptionally by the METTL3/14 complex, but its direct roles in RNA processing remain unclear. Here, we investigate the presence of m^6^A in nascent RNA of mouse embryonic stem cells. We find that around 10% of m^6^A peaks are located in alternative introns/exons, often close to 5′ splice sites. m^6^A peaks significantly overlap with RBM15 RNA binding sites and the histone modification H3K36me3. Acute depletion of METTL3 disrupts inclusion of alternative introns/exons in the nascent transcriptome, particularly at 5′ splice sites that are proximal to m^6^A peaks. For terminal or variable-length exons, m^6^A peaks are generally located on or immediately downstream from a 5′ splice site that is suppressed in the presence of m^6^A and upstream of a 5′ splice site that is promoted in the presence of m^6^A. Genes with the most immediate effects on splicing include several components of the m^6^A pathway, suggesting an autoregulatory function. Collectively, our findings demonstrate crosstalk between the m^6^A machinery and the regulation of RNA splicing.

RNA is subject to diverse post-transcriptional modifications that have emerged as new layers of gene regulation (Fu et al. 2014; Yue et al. 2015; Roundtree et al. 2017a). Among these, *N*^6^-methyladenosine (m^6^A) is the most prevalent and abundant internal RNA modification on mRNA. m^6^A was initially identified in the 1970s (Desrosiers et al. 1974; Perry et al. 1975), and the enzyme that catalyzes this modification was described in the mid-1990s (Bokar et al. 1994, 1997). Accumulating evidence suggests that RNA m^6^A modifications are, in the most part, installed by the METTL3/14 core heterodimer (Liu et al. 2014), which together with accessory proteins WTAP (Ping et al. 2014), VIRMA (Schwartz et al. 2014), RBM15/15B (Patil et al. 2016), CBLL1 (Růžička et al. 2017), and ZC3H13 (Knuckles et al. 2018; Wen et al. 2018), forms the m^6^A writer complex. Structural studies have revealed that METTL3 is the only catalytic subunit, whereas METTL14 has a degenerate active site and maintains integrity of the complex and substrate RNA recognition (Śledź and Jinek 2016; Wang et al. 2016a,b). Similar to DNA and histone modifications pathways, the m^6^A pathway has specific eraser (FTO and ALKBH5) and reader proteins (YTH-domain-containing proteins, YTHDC1/2 and YTHDF1/2/3) (Zaccara et al. 2019).

Global m^6^A patterns have been profiled using m^6^A-specific antibodies coupled to high-throughput sequencing (Meyer et al. 2012; Dominissini et al. 2013; Ke et al. 2015; Linder et al. 2015). Antibody-free m^6^A profiling methods, MAZTER-seq (Garcia-Campos et al. 2019) and m^6^A-REF-seq (Zhang et al. 2019), have been developed since but are limited to a subset of the m^6^A (m^6^ACA) sites. Extensive m^6^A profiling in a variety of RNA populations from diverse species and tissues has revealed that the majority of mRNAs are m^6^A modified with preferred sites occurring in clusters, most commonly in the 3′ UTR and around the stop codon (Dominissini et al. 2012; Meyer et al. 2012). Individual m^6^A sites have the consensus sequence DRACH (Dominissini et al. 2012; Meyer et al. 2012; Linder et al. 2015). The installation of m^6^A by the writer complex occurs cotranscriptionally, and sites are found both in exons (the majority) and introns (Ke et al. 2017; Louloupi et al. 2018). An important factor for targeting m^6^A to defined sites is the RNA-binding protein RBM15/15B, a subunit of the m^6^A writer complex (Patil et al. 2016; Coker et al. 2020). Additionally, the METTL14 subunit recognizes the histone modification H3K36me3, which is enriched within gene bodies of active genes (Huang et al. 2019). Finally, some transcription factors (TFs) have been proposed to facilitate m^6^A targeting—for example, SMAD2/3 (Bertero et al. 2018) and CEBPZ (Barbieri et al. 2017)—although only for a small number of transcripts in certain conditions and/or cell types.

The m^6^A modification has important functions in mRNA metabolism, for instance, in the regulation of RNA processing (Alarcón et al. 2015b), nuclear export (Roundtree et al. 2017b), turnover (Wang et al. 2014; Ke et al. 2017; Liu et al. 2020), and translation (Wang et al. 2015; Barbieri et al. 2017). There are, however, contradictory findings, for example, in relation to alternative splicing (Alarcón et al. 2015a; Xiao et al. 2016; Ke et al. 2017), translation and turnover (Wang et al. 2015; Lasman et al. 2020; Zaccara and Jaffrey 2020; Zhang et al. 2020), and X Chromosome inactivation (Patil et al. 2016; Nesterova et al. 2019; Coker et al. 2020). Confounding factors include the difficulty in discriminating primary and secondary effects following chronic long-term knockout/knockdown of m^6^A writers/readers, cell lethality effects linked to the important role of m^6^A in essential cell functions (Barbieri et al. 2017; Xiao et al. 2018), and cell type–specific effects. In this study, we map the intronic m^6^A methylation in mouse embryonic stem cells (mESCs) and investigate the effect of acute depletion of METTL3 on nascent RNA splicing.

## Results

### Mapping m^6^A in the nascent mESC transcriptome

**Figure 1.**
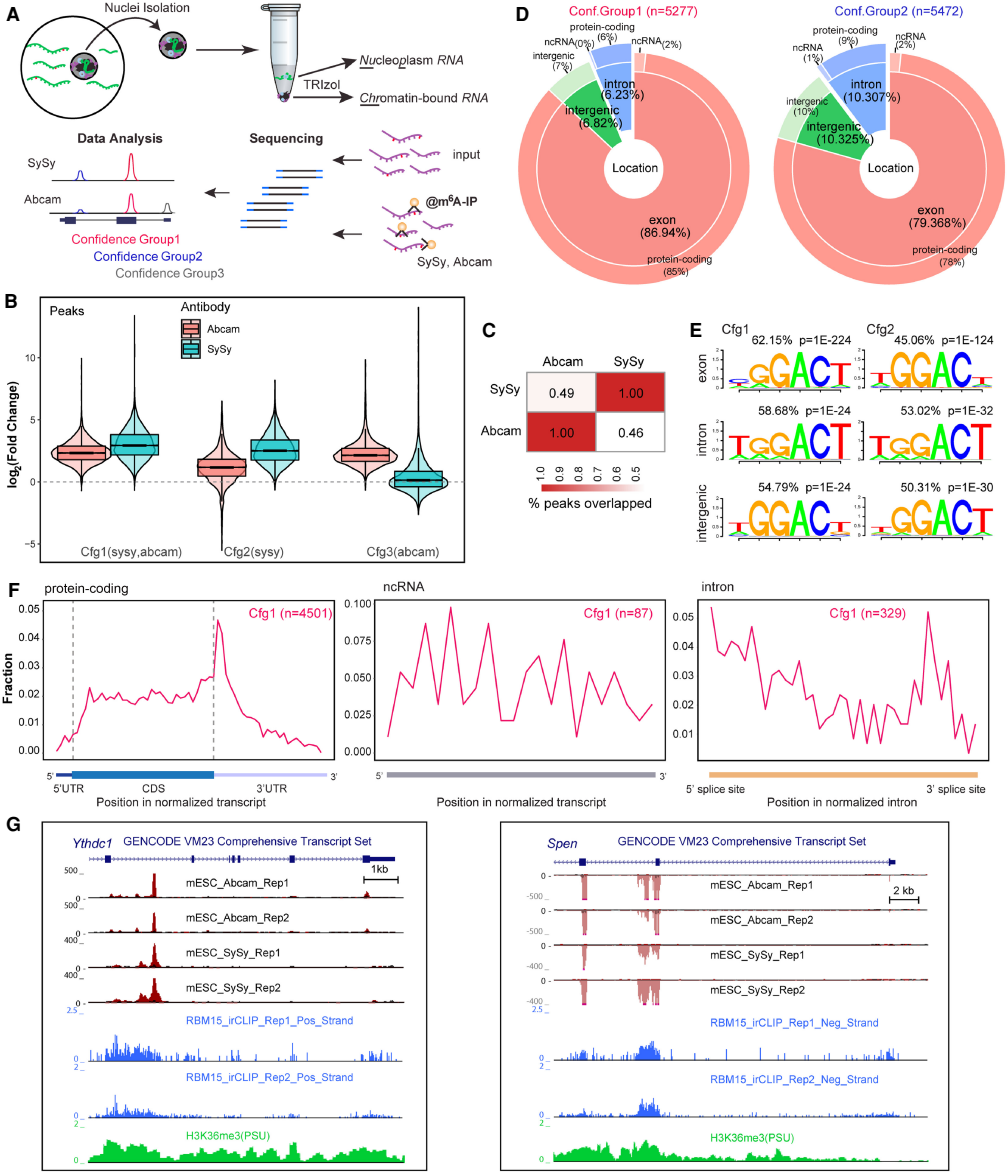
ChrMeRIP-seq reveals that 6%–10% of m^6^A peaks are located in introns. (*A*) Schematic illustrating the experimental and computational workflow for ChrMeRIP-seq. Chromatin-associated RNAs enriched for introns were used for MeRIP with two commercially available m^6^A antibodies (SySy and Abcam). Three confidence groups of m^6^A sites were identified. (*B*) Box plots showing the m^6^A intensity distributions for Confidence group (Cfg) 1, 2, and 3. Pink and cyan represent m^6^A intensity from Abcam and SySy antibodies, respectively. (*C*) Heat map showing the peak overlap between two antibodies. (*D*) Pie chart output from RNAmpp analysis showing the distribution of m^6^A peaks for Cfg1 (*left*) and Cfg2 (*right*) group. Peak numbers are indicated *above*. The MaxORF and longestNcRNA isoform was chosen for each gene. (*E*) Most representative motifs called for each subgroup (exonic, intronic, and intergenic) in Cfg1 and Cfg2 groups. Peak numbers are indicated. (*F*) RNAmpp analysis of m^6^A peaks distributions in transcriptome for Cfg1 group. *Left* plot is for aggregated protein-coding gene, *middle* for noncoding RNA, and *right* for normalized intron. (*G*) UCSC Genome Browser screenshots showing example genes (*left*, *Ythdc1* intron11; *right*, *Spen* intron2) harboring intronic m^6^A methylation. From *top* to *bottom*, tracks denote gene annotation, ChrMeRIP-seq (Abcam 2 replicates, SySy 2 replicates), RBM15 irCLIP-seq (two replicates), and H3K36me3 ChIP-seq. The red and black in ChrMeRIP-seq indicate the IP and input, respectively.

Chromatin-associated RNA (ChrRNA) is substantially enriched for nascent transcripts (Nesterova et al. 2019). Thus, to investigate the roles of m^6^A in nascent RNA processing in mouse embryonic stem cells, we performed MeRIP-seq from ChrRNA, referred to henceforth as ChrMeRIP-seq (Fig. 1A; Supplemental Fig. S1A; Supplemental Table S1). Sequencing of input showed that ∼70%–80% of reads are intronic (Supplemental Fig. S1B). To minimize specific antibody bias, we used two commercially available m^6^A antibodies (SySy and Abcam) to identify high-confidence m^6^A-modified RNA regions. Using maximum ORF and longest ncRNA isoforms as representative transcripts (see Methods), refined peak calling analysis (see Methods) classified 5277, 5472, and 6319 m^6^A peaks into Confidence group1 (high), Confidence group2 (medium), and Confidence group3 (low), respectively (Fig. 1B–D; Supplemental Fig. S1C,D; Supplemental Data S1). The trend of m^6^A peak intensity in the different groups accords with their confidence classifications (Fig. 1B; Supplemental Fig. S1E). The overlap between peaks from SySy and Abcam antibodies is approximately half, which is similar to the differences in peak detection surveyed between studies (Fig. 1C; McIntyre et al. 2020). Despite the large fraction of intronic reads in the input, only 6.2% of Confidence group1 (Cfg1) and 10.3% of Confidence group2 (Cfg2) peaks are from intronic regions (Fig. 1D; Supplemental Fig. S2A), as defined by the position of the single-nucleotide peak summit (see Methods). This is slightly higher than previously reported for MeRIP-seq or m^6^A-CLIP studies using only messenger RNA from mESCs (Supplemental Fig. S2B; Batista et al. 2014; Geula et al. 2015; Ke et al. 2017) and is in line with ChrMeRIP-seq from HeLa cells (Ke et al. 2017). The majority of intronic m^6^A modification occurs in protein-coding genes rather than noncoding RNAs (Fig. 1D; Supplemental Fig. S2A,B).

We developed an RNA metaprofile plot (RNAmpp) to describe the distribution of m^6^A in the nascent transcriptome (see Methods). m^6^A peaks from both confidence groups 1 and 2 are enriched around stop-codon regions or at the beginning of the 3′ UTR in mRNAs. For all m^6^A peak genomic categories, the canonical DRACH m^6^A motif (GGACU) is the most highly represented motif (Fig. 1E,F; Supplemental Fig. S2C,D). The few m^6^A peaks which map to lncRNAs are not close to the 3′-end regions but are distributed randomly across the transcripts, as exemplified by *Xist*, *Norad*, and *Malat1* lncRNA genes (Supplemental Fig. S2E; Patil et al. 2016; Coker et al. 2019). We found several clear examples of intronic m^6^A peaks located close to 5′ splice sites, such as the two intronic m^6^A peaks from *Ythdc1* intron 11 and *Spen* intron 2 (Fig. 1G).

**Figure 2.**
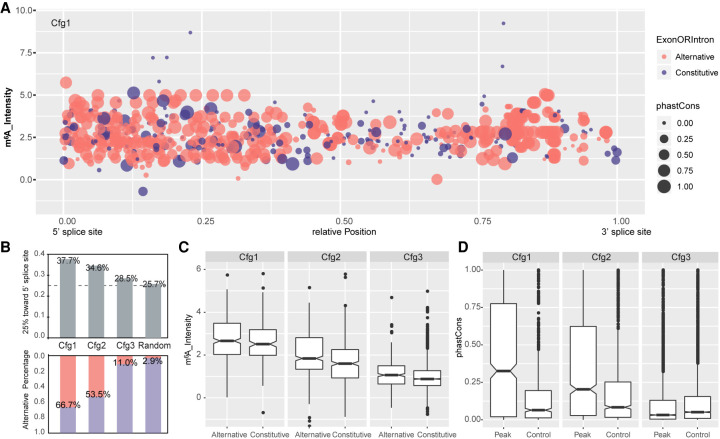
Patterns of intronic m^6^A modification. (*A*) Dot plots show the relative position, m^6^A intensity, conservation, and location in alternative or constitutive introns for all intronic m^6^A methylation in Cfg1. Here, 0 and 1 in the *x*-axis represent the 5′ and 3′ splice sites, respectively. The *y*-axis denotes m^6^A intensity calculated as an average of all replicates. Dot area indicates the phastCons conversation score. Red and blue denote location in alternative and constitutive introns, respectively. (*B*) Bar plots (*top*) showing the fraction of m^6^A peaks located in the first quarter (close to the 5′ splice site) of introns for Cfg1, Cfg2, and Cfg3 classes, as well as random simulated peak summits. Bar plots (*bottom*) showing the fraction of m^6^A peaks located in alternative exon/introns for all the groups. The percentages for each bar are labeled. (*C*) Box plots showing intensity of intronic m^6^A peaks located in alternative and constitutive introns for all classes. (*D*) Box plot of phastCons scores for all classes of m^6^A peaks located in intron regions annotated from the MaxORF_LongestNcRNA isoforms, compared with controls matched for size and intron-of-origin.

Given that the pattern of exonic m^6^A has been extensively characterized (Dominissini et al. 2012; Meyer et al. 2012), we sought to specifically investigate the deposition pattern and characteristics of intronic m^6^A modification. To reduce bias, we compared the GC content, conservation level, and relative position of intronic m^6^A peaks to their size-matched control regions derived from random regions within the same intron (see Methods). This analysis shows that intronic m^6^A methylations are more common in regions which have high GC content, are evolutionarily conserved, and are in proximity to 5′ splice sites (Fig. 2A–D; Supplemental Fig. S3A–C). When compared to randomly chosen introns from the same genes as control, longer introns are preferentially methylated (Supplemental Fig. S3D).

Most exonic methylations are enriched around stop-codon regions, as previously noted (Dominissini et al. 2012; Meyer et al. 2012), but intronic methylation sites are fairly evenly distributed relative to host transcripts (Supplemental Fig. S3E). Therefore, we queried in particular whether intronic methylated regions are located close to alternative exons or introns using the comprehensive GENCODE annotation set (vM24). Indeed, we found that higher confidence groups of m^6^A-methylation were more likely to reside in alternative intron/exon regions (see Methods) than low-confidence groups or random genomic regions (Fig. 2A,B). This indicated a potential role of the METTL3 complex and its methylation sites in the regulation of alternative splicing.

### Intronic m^6^A modifications correlate with RBM15 binding and H3K36me3

The RNA-binding protein RBM15 plays an important role in targeting m^6^A to defined sites in mRNA (Patil et al. 2016). We went on to examine if this pathway is linked to the intronic m^6^A sites that we observe in nascent RNA. To map binding sites for RBM15 in mESCs, we performed infrared cross-linking immunoprecipitation followed by sequencing (irCLIP-seq) (Zarnegar et al. 2016), making use of an mESC line in which both *emGFP-Rbm15* and *Xist* RNA are induced by treatment with doxycycline (Supplemental Fig. S4A; Coker et al. 2020). RBM15 interacts with the *Xist* A-repeat region and contributes to the deposition of m^6^A methylation at sites immediately downstream (Supplemental Fig. S4B; Patil et al. 2016; Coker et al. 2020) and thus provides a useful positive control.

**Figure 3.**
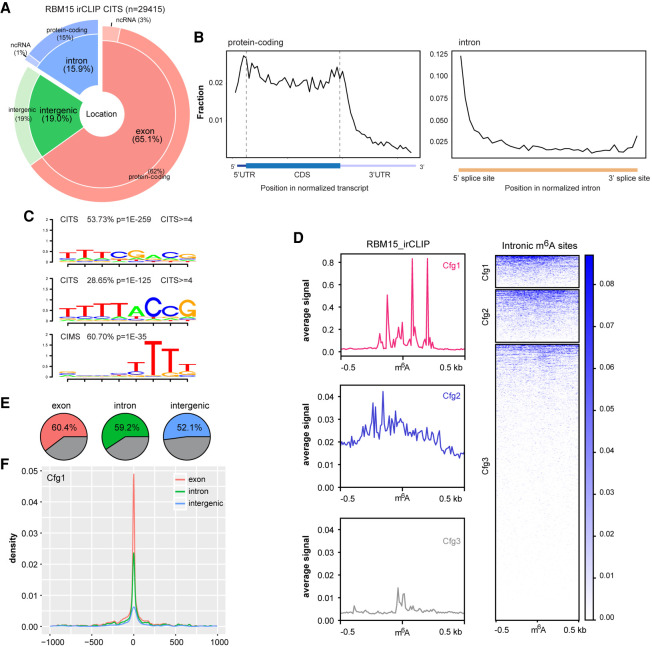
Intronic m^6^A methylation correlates with RBM15 binding and H3K36me3. (*A*) Pie chart shows the distribution of RBM15 binding sites, calculated from irCLIP cross-linking induced truncation sites (CITS ≥3). (*B*) The RNA-binding profiles of RBM15 in the transcriptome, calculated for aggregated gene models for protein-coding genes (*left*) and introns (*right*). (*C*) RNA-binding motifs occurring at RBM15 CITS and CIMS. (*D*) The RBM15 binding (CITS) metaprofile and heat map for intronic m^6^A peaks of three different confidence groups (red, blue, and gray for Cfg1, Cfg2, and Cfg3, respectively). The color key is shown on the *right*; 0.5-kb strand-specific flanking regions on each side of m^6^A peak summits are included for the plot. (*E*) Pie charts illustrating the fraction of Cfg1 m^6^A peaks with strong RBM15 CITS (≥3) within 1-kb flanking regions. Exonic, intronic, and intergenic m^6^A peaks are shown from *left* to *right*. (*F*) The RBM15 binding sites distribution (CITS≥3) centered on m^6^A peaks. Red, green, and blue lines represent exonic, intronic, and intergenic m^6^A peaks from Cfg1, respectively.

Cross-linking induced truncation sites (CITSs or RT stops) are the main signature occurring in irCLIP-seq data sets. Use of the RNAmpp analysis shows where these CITSs reside across normalized transcripts, with two main peaks at the start of the transcript and near the stop-codon region (Fig. 3A,B), in agreement with the RBM15/15B binding profile in human cells (Patil et al. 2016). Examination of RBM15 binding across introns shows a preference for the 5′ splice site, consistent with the profile of intronic m^6^A (Fig. 3B; Supplemental Fig. S4C). Motif analysis of CITSs revealed an RBM15 binding consensus comprising three or four consecutive U bases, both for exonic and intronic sites (Fig. 3C; Supplemental Fig. S4D). This is also the case for cross-linking-induced mutations (CIMS) (Fig. 3C). We found that RBM15 binding is centered at m^6^A peak summits for all exonic, intronic, and intergenic regions and generally correlates with peak confidence (Fig. 3D; Supplemental Fig. S5A,B).

We further split each m^6^A confidence group by strong or weak RBM15 binding, based on whether strong RBM15 binding sites (CITS ≥3 in two replicates) intersected the m^6^A peak. Overall, approximately half of the sites in all subgroupings except Cfg3 were assigned as strong RBM15 binding (Fig. 3E,F; Supplemental Fig. S5C–F). RBM15 binding was centered on m^6^A peaks in all of the confidence groups (Fig. 3F; Supplemental Fig. S5D,F). In addition to RBM15, the histone modification H3K36me3 has been proposed to play a role in directing m^6^A to defined sites in mRNA, mediating interaction with the METTL14 subunit of the core m^6^A complex (Huang et al. 2019). Consistently, we observed that high-confidence exonic and intronic m^6^A peaks correlate with higher H3K36me3 density (Supplemental Fig. S6A,B). Furthermore, m^6^A peaks with strong RBM15 binding also reside within high H3K36me3 regions for both exonic and intronic sites (Supplemental Fig. S6C–E). Taken together, our observations indicate that intronic m^6^A sites show equivalent correlations with both RBM15 binding and H3K36me3 density to those seen for exonic sites, suggesting that similar targeting mechanisms function in both contexts.

### Rapid depletion of METTL3 using the dTAG system

**Figure 4.**
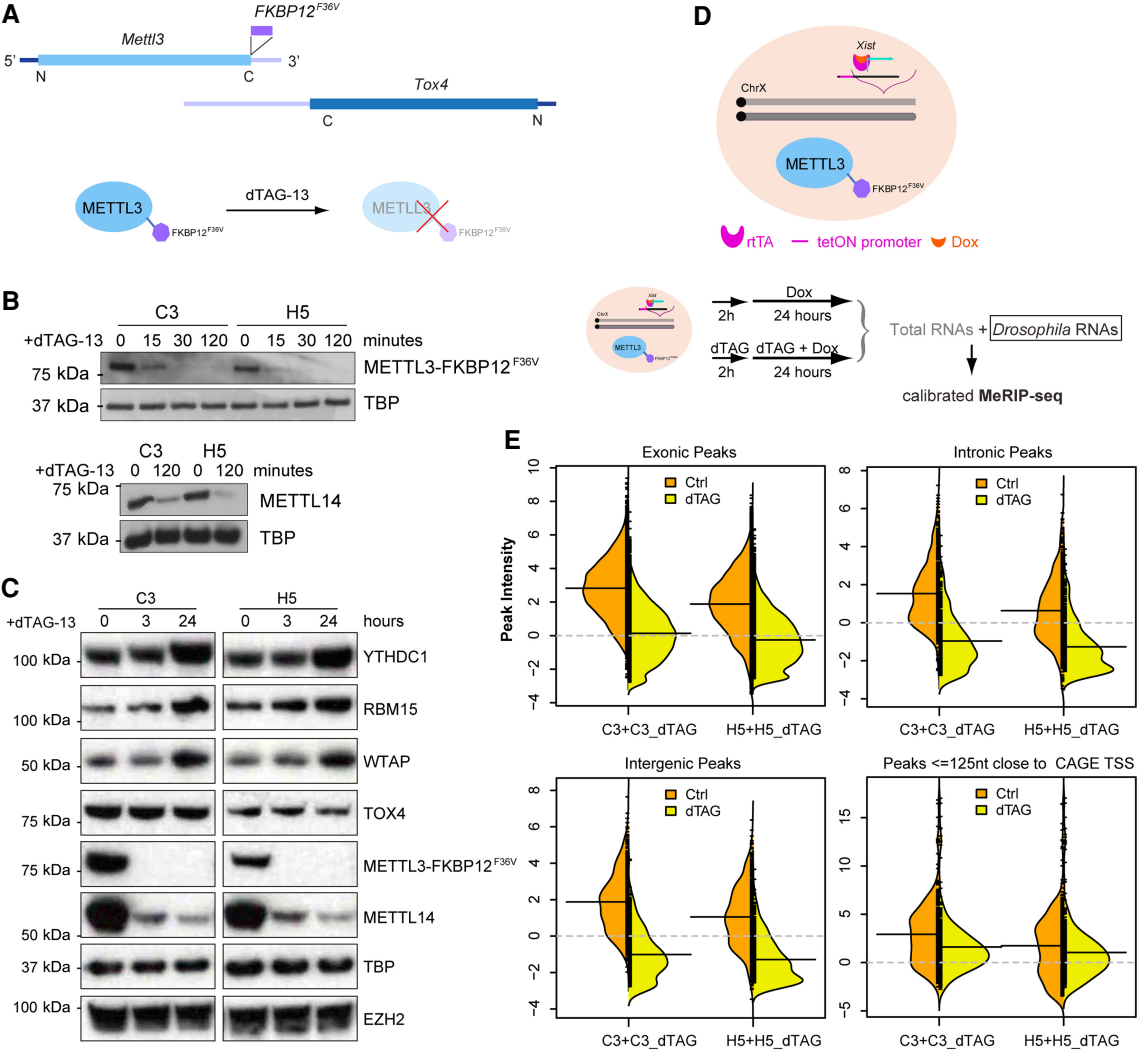
Acute depletion of METTL3 by dTAG system. (*A*) Schematic illustrates the *FKBP12*^*F36V*^ insertion into the stop codon of the *Mettl3* gene, which overlaps with *Tox4* in an antisense manner. dTAG-13 molecules engage FKBP12^F36V^ to trigger degradation of the fusion protein. (*B*) Western blots show degradation of METTL3_FKBP12^F36V^ in a dTAG time-course treatment experiment (15, 30, 120 min) in two independent clones C3 and H5 (*top*). *Lower* panel shows METTL14 protein levels in C3 and H5 clones upon 120-min dTAG-13 treatment. TBP acts as a loading control. (*C*) Western blots show the protein levels for YTHDC1, RBM15, WTAP, TOX4, together with METTL3_FKBP12^F36V^ and METTL14 in C3 and H5 clones upon 3- or 24-h dTAG-13 treatment. TBP and EZH2, encoded by another non-m^6^A-modified RNA, serve as loading controls. (*D*) Schematic showing *FKBP12*^*F36V*^ inserted into the *Mettl3* locus of hybrid XX mESCs expressing doxycycline-inducible *Xist*, and the calibrated MeRIP-seq workflow with *Drosophila* RNAs as a spike-in (*bottom*). *Xist* was induced after 2-h dTAG-13 treatment. (*E*) Bean plots of the calibrated m^6^A intensity distributions for peaks classified as exonic, intronic, intergenic from SySy antibody, as well as peaks within 125 nt of CAGE TSS. *Left* and *right* bean plots show clones C3 and H5, respectively. Orange and yellow back-to-back plots represent Ctrl and dTAG, respectively. The black solid lines denote the mean of each distribution, and gray dashed lines represent the threshold of nonenrichment.

Functional analysis of the METTL3/14 m^6^A writer complex using gene knockout/knockdown has provided conflicting results in terms of the importance of m^6^A in regulating splicing. A confounding factor is that m^6^A has roles in mRNA stability and translation (Fu et al. 2014), and it is challenging to discriminate primary and secondary effects resulting from chronic or incomplete loss of function. To address this, we developed an acute METTL3 knockout model using the dTAG degron system (Nabet et al. 2018). *FKBP12*^*F36V*^ was fused in-frame into the C-terminus of *Mettl3* in female mESCs with doxycycline-inducible *Xist* using CRISPR-Cas9–mediated knock-in (Fig. 4A). In two independent clones (C3 and H5), the expression level and subcellular localization of METTL3_FKBP12^F36V^ were very similar to those of endogenous METTL3. We also confirmed that the *FKBP12*^*F36V*^ insertion does not interrupt the protein level of the neighboring *Tox4* gene, whose 3′ UTR locus overlaps with the last two coding exons of *Mettl3* in an antisense manner (Fig. 4A,C). Following treatment with dTAG-13, METTL3_FKBP12^F36V^ protein levels were rapidly depleted, within 30 min (Fig. 4B). We also observed a strong reduction in levels of METTL14, which forms a stable heterodimer with METTL3, suggesting heterodimer formation is important for METTL14 protein stability (Fig. 4B,C; Supplemental Fig. S7A).

We next sought to examine global dependence of m^6^A on the METTL3 complex by performing calibrated MeRIP-seq (Zeng et al. 2018) after 26 h dTAG-13 treatment (Supplemental Table S2). *Xist* RNA, a useful indicator for m^6^A deposition (Ke et al. 2015; Linder et al. 2015; Patil et al. 2016; Nesterova et al. 2019; Coker et al. 2020), was induced after 2 h dTAG-13 treatment (Fig. 4D). For untreated cells, m^6^A peaks were found at previously annotated sites including *Xist* RNA (Nesterova et al. 2019), indicating that METTL3_FKBP12^F36V^ retains functionality for m^6^A modification deposition (Supplemental Fig. S7B,C). Following dTAG-13 treatment, most *Xist* m^6^A peaks were undetectable, including the characteristic sites downstream from the *Xist* E-repeat (Supplemental Fig. S7B,C). Moreover, the majority of exonic, intronic, and intergenic m^6^A peaks became indistinguishable in intensity from input (Fig. 4E; Supplemental Fig. S7C,D). The only exceptions were peaks that are close to transcript start sites (TSSs), including in *Xist* RNA, which likely represent m^6^Am modification installed by PCIF1 (Akichika et al. 2019) rather than METTL3/14 (Fig. 4E; Supplemental Fig. S7B–D). Collectively, these results demonstrate that the dTAG system enables rapid depletion of METTL3 and m^6^A in mRNA.

### Acute depletion of METTL3 reveals a role for m^6^A in alternative splicing

**Figure 5.**
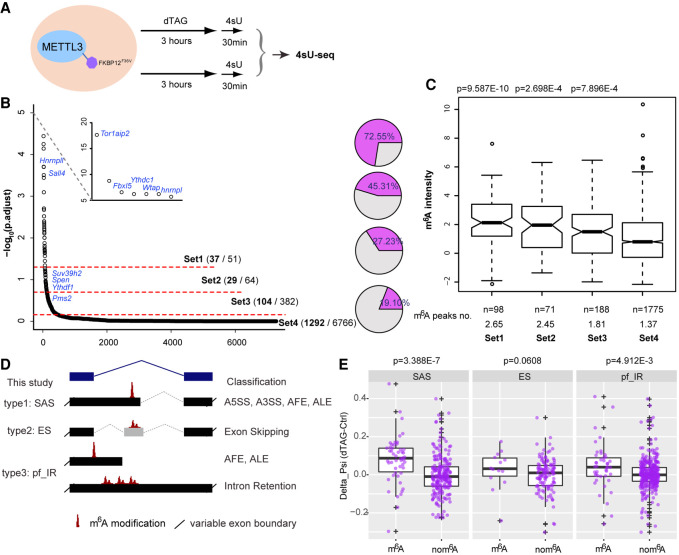
Rapid depletion of METTL3 causes m^6^A-targeted alternative splicing. (*A*) Schematic of 4sU-seq experimental design. (*B*) The output from intron-centric software LeafCutter ranks splicing clusters that change upon dTAG-13 treatment. Splicing clusters are ordered on the *x*-axis according to their significance, which is plotted on the *y*-axis (−log_10_[p.adjust]). Three different cutoffs were set to produce four groups graded by splicing significance. Selected genes are labeled. Pie charts (*right*) show the fraction of splicing cluster having or neighboring m^6^A modification. (*C*) Box plots showing the m^6^A intensity distribution calculated from ChrMeRIP-seq. m^6^A peaks from high to low significance groups are ordered from *left* to *right*. *P*-values *above* boxes were calculated by a two-sided *t*-test for each group with respect to the non-splicing-change group (far *right*). The total peak number and average peak intensity for each splicing cluster are shown *below*. (*D*) Schematic showing types of splicing classified, with nomenclature used in this study (*left*) and canonical splicing classification (*right*). (*E*) Box plots comparing the deltaPSI for each group (SAS, ES, and pf_IR). Splicing clusters with m^6^A modification located at the alternative intron/exon (*left*) are compared to splicing clusters from same group without m^6^A modification (*right*) as a batch-matched control. *P*-values shown *above* are calculated by a one-sided Wilcoxon test. Positive deltaPSI indicates increased inclusion upon depletion of METTL3.

We went on to examine the effect of METTL3 depletion on splicing by analyzing the newly synthesized transcriptome using 4sU-seq after dTAG-13 treatment for 3 h, followed by a short 4sU incorporation (30 min) (Fig. 5A). This enabled us to explore transcriptional or cotranscriptional changes upon depletion of METTL3 while limiting indirect effects from m^6^A-mediated RNA destabilization (Wang et al. 2014; Liu et al. 2020). We validated that mRNA destabilization effects were minimal by performing differential gene expression analysis. Only a few differentially regulated genes were found (Supplemental Fig. S8; Supplemental Data S2), compared with thousands observed after long-term *Mettl3* knockout/knockdown (Batista et al. 2014; Geula et al. 2015; Yue et al. 2015; Ke et al. 2017). We then employed LeafCutter to perform intron-centric annotation-free differential splicing analysis (Li et al. 2018). The splicing events were sorted into four groups by graded significance, with Set1 as the most splicing changed group (Supplemental Data S3). The higher the significance cutoff, the higher was the proportion of differential splicing events that include or neighbor m^6^A peaks, with 72.5% in Set1 with the most significant threshold, and only 19% in Set4 with the lowest threshold (Fig. 5B). Consistent with this, the m^6^A peak intensities and peak numbers overlapping Set1 splicing clusters were significantly higher than the remaining groups (Fig. 5C). These analyses of the early response in the nascent transcriptome imply that the METTL3 complex affects the inclusion of specific splicing elements, potentially by depositing m^6^A modifications at nearby splice sites.

We next focused on characterizing alternative splicing defects caused by METTL3 depletion using the aforementioned Set1-3 clusters. We grouped the intron-centric alternative splicing clusters from the LeafCutter output into three types: (I) Splice Alternative Site (SAS); (II) Exon Skipping (ES); and (III) partial or full Intron Retention (pf_IR) (Fig. 5D and Methods). To avoid batch effects, significant splicing changes occurring at sites without m^6^A modification from the same sample and the same splicing type were chosen as matched controls (Fig. 5B,E). To compare results for types I–III, we used the splicing changes (deltaPSI) upon dTAG-13 treatment for the splicing form which has longer introns and skips the alternative splicing element as reference (Fig. 5D and Methods). This is because pf_IR (type III) does not contain other splicing events within the reference splicing form. The deltaPSI of the reference splicing forms are always reciprocal to changes of the alternative splicing forms that bear m^6^A modifications (Fig. 5D,E). This analysis shows that, for all the described splicing types, the reference splicing forms are increased compared to their controls following dTAG-13 treatment, most significantly for SAS and pf_IR types (Fig. 5E).

**Figure 6.**
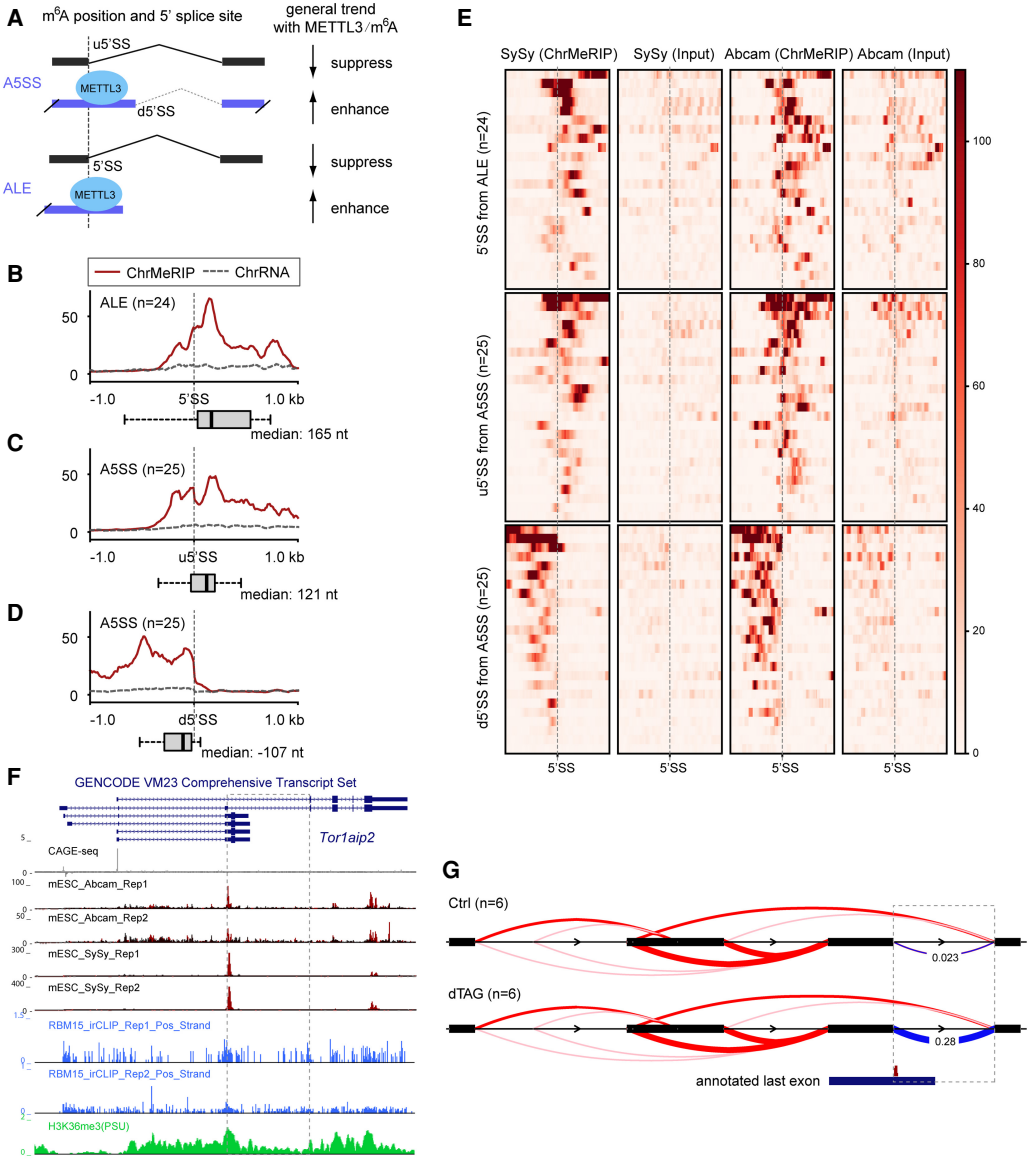
METTL3/m^6^A-mediated alternative intron/exon inclusion. (*A*) Schematic showing the general splicing output for splicing type A5SS and ALE with m^6^A modification. (*B*–*D*) Aggregate m^6^A signals over the 5′ splice sties (±1 kb) for ALE (*n* = 24) (*B*) and upstream (*C*) or downstream (*D*) 5′ splice site for A5SS (*n* = 25). Red solid and dashed gray lines indicate the ChrMeRIP and ChrRNA input samples, respectively. Box plots denote the distance distribution between the 5′SS and the closest m^6^A peak summit. ChrMeRIP and input samples are from SySy. *y*-axis shows normalized intensity. (*E*) Heat map showing (ChrMeRIP and Input of SySy and Abcam) signal intensity for each 5′ splice site as well as strand-specific flanking 1-kb region from splicing changed A5SS and ALE from *B*–*D*. Gray dashed lines indicate 5′ splice sites for each type. (*F*) Genome Browser tracks for *Tor1aip2* gene. From *top* to *bottom*, they denote CAGE-seq, ChrMeRIP-seq (Abcam, two replicates; SySy, two replicates), RBM15 irCLIP-seq (two replicates), and H3K36me3 ChIP-seq. The dashed box depicts the most significantly changed splicing cluster in this study. (*G*) Sashimi plot of the *Tor1aip2* splicing cluster. The dashed box is the same as *F* with deltaPSI calculated from LeafCutter. The annotated last exon containing m^6^A modification at the splicing site is shown *below*.

When transcriptional direction was considered in the m^6^A-linked splicing events, we found that two-thirds (33 out of 50) of SAS events have alternative 5′ splice sites and three-quarters (33 out of 43) of pf_IR events overlap with 5′ splice sites, which agrees with the nascent m^6^A pattern that intronic m^6^A modifications are generally in proximity to 5′ splice sites. Although both SAS and pf_IR events are associated with alternative intron/exon inclusion, in general we found that, for SAS events, splicing of downstream 5′ splice sites (d5′SS) is enhanced and splicing of upstream 5′ splice sites (u5′SS) is repressed in the presence of METTL3 in A5SS cases (25 out of 33), whereas in pf_IR events, inclusion of the intron-derived alternative last exon (ALE) is enhanced in the presence of METTL3 (24 out of 33) (Fig. 6A).

With these observations in mind, we sought to determine if m^6^A position could account for the aforementioned splicing outputs (Fig. 6A). For this analysis, we have assumed that m^6^A affects nearby splice sites in *cis*. The distance between 5′ splice sites and the corresponding closest m^6^A peak summit was calculated accordingly. For both the 5′SSs in ALE and the u5′SSs in A5SS that are suppressed in the presence of METTL3, we found that m^6^A peaks are either overlapped with or located downstream from the 5′ splice sites (Fig. 6B,C,E; Supplemental Fig. S9A). In contrast, m^6^A peaks are located upstream of the enhanced d5′SSs in A5SS (Fig. 6D,E; Supplemental Fig. S9A). A similar pattern was also seen for RBM15 binding (Supplemental Fig. S9B). This indicates that METTL3-mediated m^6^A deposition directly decreases the capacity of spliceosomes to recognize the 5′SS in ALE and the u5′SS in A5SS, which leads to inclusion of ALE or use of the d5′SS, respectively. We next sought to explore the relationship between m^6^A density and effect size of alternative splicing changes (deltaPSI) and found that m^6^A intensity at alternative 5′ splice sites (A5SS) correlates with the observed splicing changes (Supplemental Fig. S9C,D). Moreover, the splicing changes of these m^6^A-bearing alternative 5′-splicing events observed in dTAG METTL3 phenocopy those seen in *Ythdc1* conditional knockout (Supplemental Fig. S10; Liu et al. 2020). Together, these findings suggest positioning of m^6^A in introns relative to the 5′ splice sites may provide a basis for specifying alternative splicing outcomes.

We went on to compare our findings with those from stable *Mettl3* knockout mESCs (data sets from Ke et al. 2017 and Geula et al. 2015). Applying a similar pipeline, we found that only 25%–34% of the splicing clusters include or neighbor our annotated m^6^A peaks, and this percentage decreases alongside the m^6^A peak number per splicing cluster from Set1 to Set4 (Supplemental Fig. S11A–F). Furthermore, the nascent splicing changes upon acute depletion of METTL3 barely overlapped with the splicing changes observed in the mature transcriptome from constitutive *Mettl3* knockout (Supplemental Fig. S11G; Geula et al. 2015; Ke et al. 2017), indicating that secondary effects on splicing are dominant in stable *Mettl3* knockout mESCs. We did, however, find evidence that *Mettl3* knockout has a small effect on m^6^A-containing cassette exon skipping, as reported (Supplemental Fig. S11E,F; Xiao et al. 2016). This predominance of secondary effects is not surprising given that *Mettl3* knockout affects a variety of RNA processing steps such as nuclear export (Roundtree et al. 2017b) and RNA decay (Wang et al. 2014; Ke et al. 2017; Liu et al. 2020).

Intron3 of the *Tor1aip2* gene is the most strongly affected differentially spliced gene in our acute METTL3 depletion experiment (Fig. 5B) and also ranks among the top four in two previous *Mettl3* knockout studies (Supplemental Fig. S11A,C; Geula et al. 2015; Ke et al. 2017). m^6^A modification renders the short isoform dominant under normal conditions, whereas the longer isoform (alternative last exon) becomes extensively induced, with loss of m^6^A significantly increasing the splicing at this junction (Fig. 6F,G; Supplemental Fig. S12). Similar results reported to occur with conditional knockout of the nuclear m^6^A reader *Ythdc1* in mESC (Liu et al. 2020) further demonstrate the role of m^6^A as opposed to other functions of the METTL3/14 complex in regulating splicing (Supplemental Fig. S12B). We next queried the relationship between this intron splicing and m^6^A levels. In a previous study, we observed that knockout of different subunits of the m^6^A writer complex (*Mettl3*, *Wtap*, and *Rbm15*) results in different residual m^6^A levels (Nesterova et al. 2019). Note that previously described *Mettl3* knockouts do not entirely deplete m^6^A, likely because the mutant alleles are hypomorphic and/or there are other m^6^A methyltransferases such as METTL16. By exploring ChrRNA-seq generated from the aforementioned knockouts, we found that the occurrence of intron splicing correlates with residual m^6^A levels (Supplemental Fig. S12C,D). In summary, these results indicate that m^6^A can function to mediate inclusion of m^6^A-containing alternative introns/exons in the context of the nascent transcriptome.

### Auto-regulation of the m^6^A machinery by alternative splicing

**Figure 7.**
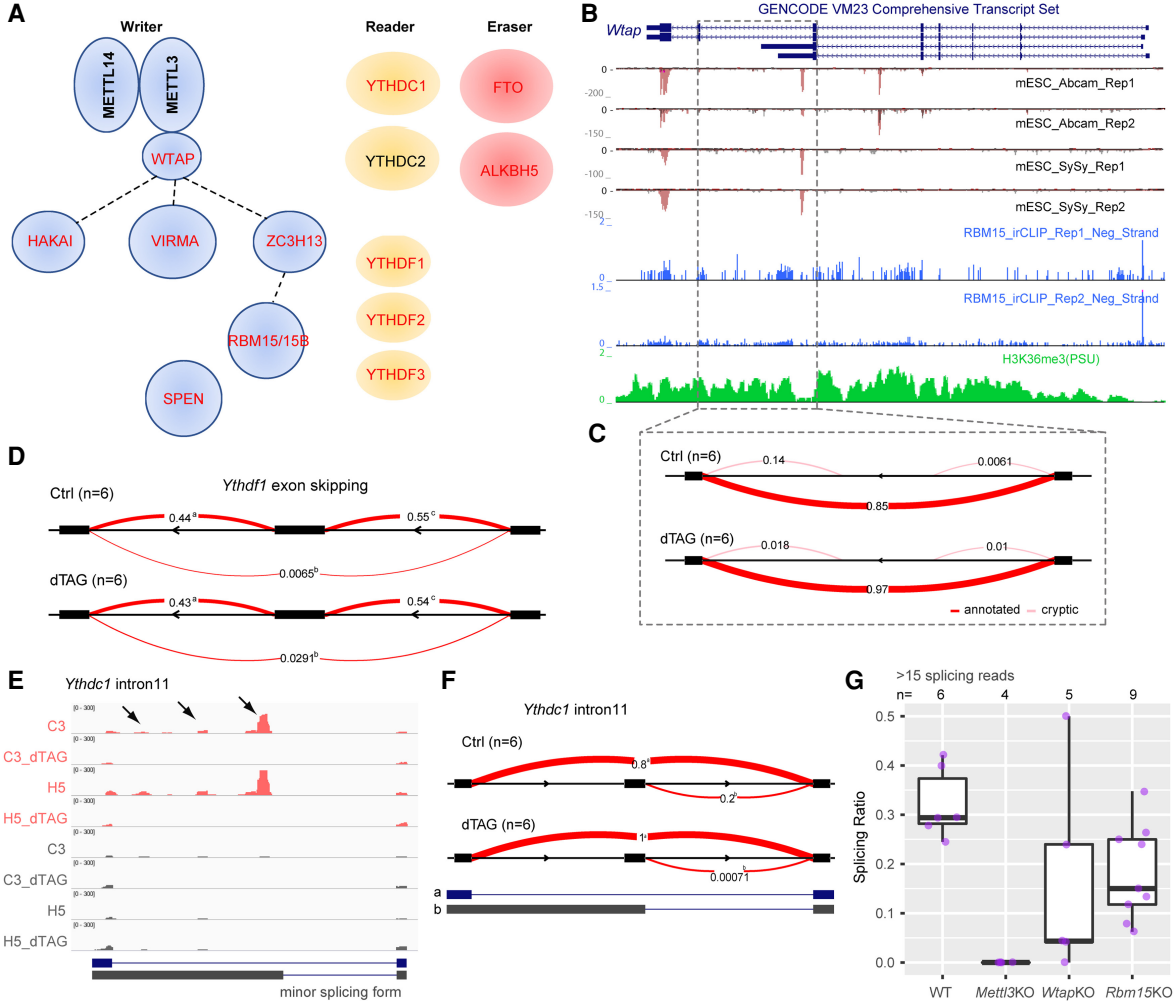
Splicing changes contribute to m^6^A self-regulation as an early consequence of acute m^6^A loss. (*A*) Schematic of m^6^A writer, reader, and eraser complexes. Genes containing m^6^A modification are indicated by red text. Dashed lines in the writer complex indicate biochemically uncharacterized interactions. (*B*,*C*) Genome Browser tracks (*B*) and Sashimi plot showing the detalPSI (*C*) for *Wtap*. Like the *Tor1aip2* gene shown in Figure 6, the splicing is of type “pf_IR.” (*D*) Sashimi plot showing the deltaPSI calculated from LeafCutter for *Ythdf1* gene, ES type. (*E*) IGV tracks showing MeRIP-seq signal (*top* four tracks) and input signal (*bottom* four tracks) for *Ythdc1* intron11. Arrowheads indicate m^6^A peaks located in the alternative intron/exon part. Annotated splicing forms are shown *below*. (*F*) Sashimi plot showing changes for *Ythdc1* intron11, which is of type “SAS.” (*G*) Box plots showing the splicing choice score for the minor splicing form of *Ythdc1* intron11 from ChrRNA-seq data sets in which components of the m^6^A writer complex are perturbed (Nesterova et al. 2019). Samples were included only if more than 15 reads span the junction at *Ythdc1* intron11.

Among thousands of m^6^A targets in mESCs, we found that all of the m^6^A cytosolic reader genes *Ythdf1/2/3* are site-specifically modified by m^6^A in their internal long CDS-coding exons (Supplemental Fig. S13A–C). *Ythdc1* encoding the nuclear m^6^A reader YTH-containing protein is heavily methylated by m^6^A across exon11 and intron11 regions (Fig. 1G). Transcripts from genes encoding accessory proteins of the core m^6^A heterodimer writer complex including *Wtap*, *Virma, Cbll1*, and *Rbm15/15B* are extensively methylated, as well as two m^6^A erasers (*Fto* and *Alkbh5*) (Fig. 7A; Supplemental Fig. S13D–F). Additionally, *Spen*, encoding an RRM and SPOC-domain-containing protein in the same family as RBM15 that has been implicated in m^6^A regulation (Dossin et al. 2020), is also heavily m^6^A-methylated across exonic and intronic regions (Fig. 1G; Supplemental Fig. S14A,B). Together, these results suggest the existence of feedback loops for regulating cellular mRNA m^6^A metabolism. Of the aforementioned examples, *Wtap*, *Ythdc1*, *Ythdf1*, and *Spen* all show m^6^A-dependent regulation of alternative splicing as an early response to m^6^A loss. The splicing changes occurring at these gene loci are of different types: *Ythdc1* intron11 and *Spen* intron2 are SAS (alternative 5′ splice site) and *Wtap* intron6 is pf_IR (alternative last exon) (Fig. 7B,C; Supplemental Fig. S15), whereas the *Ythdf1* cassette exon is ES (exon skipping) (Fig. 7D). Splicing of *Ythdc1* intron11 and *Wtap* intron6 are also significantly changed and rank as top hits in both stable *Mettl3* knockout data sets (Supplemental Fig. S11A,C; Geula et al. 2015; Ke et al. 2017).

For these examples, a splicing choice score was calculated for the minor splicing forms (alternative 5′ splice site) (see Methods). The splicing choice score for the *Ythdc1* intron11 minor splicing junction is approximately 20%–30% in wild-type cells, whereas it drops to nearly 0 in both acute and stable *Mettl3* knockout cells (Fig. 7E–G). This suggests that m^6^A modifications determine the inclusion of this minor splicing form. This is unlikely to be the consequence of RNA destabilization by m^6^A modification, which would result in the minor splicing form being overrepresented. When we explored ChrRNA-seq data sets generated from different knockouts of the m^6^A writer complex in mESCs (Nesterova et al. 2019), we observed that representation of the minor splicing forms is significantly reduced (Fig. 7F) and that the splicing effects correlate with the residual m^6^A levels caused by different knockouts (Fig. 7G). As it is the major splicing form of *Ythdc1* that produces the main protein-coding isoform, loss of m^6^A modification at intron11 (and thus the alternatively spliced short isoform) contributed to more efficient *Ythdc1* transcript production. Accordingly, the *Ythdc1* transcript (Supplemental Fig. S8A) and protein levels (Fig. 4C) are higher in the acute METTL3 knockout mESCs. Similar results were also obtained for *Spen* intron2 (Supplemental Fig. S14C) and *Wtap* intron6 (Figs. 4C, 7C; Supplemental Fig. S15). Taken together, splicing changes occurring as the immediate consequence of loss of m^6^A contribute to m^6^A self-regulation.

## Discussion

Our analysis reveals that, in mESCs, ∼6%–10% of high-confidence m^6^A regions are located in introns, in broad agreement with a prior analysis of nascent RNA from HeLa cells (Ke et al. 2017). We observe preferential location of intronic m^6^A close to 5′ splice sites. Both intronic and exonic m^6^A regions show dependence on the METTL3/14 writer complex, as determined by acute depletion of METTL3 using the dTAG system. Our data further indicate that intronic m^6^A modifications are deposited through the same mechanisms as those reported to function in exons, RBM15 binding and H3K36me3-modified chromatin (Patil et al. 2016; Huang et al. 2019). Of note, we observed preferential RBM15 binding both around the stop codon/3′ UTR and at the start of transcripts, with only the former correlating with the distribution of m^6^A. Similar binding profiles were observed in a prior study analyzing human RBM15/15B protein (Patil et al. 2016). The basis for preferred binding near the start of transcripts is currently unknown but may be linked to interaction of the RBM15 SPOC domain with the H3K4me3 methyltransferase SET1B that localizes to gene promoters (Lee and Skalnik 2012; Coker et al. 2020). Accordingly, RBM15 binding close to the start of transcripts, which does not correlate with m^6^A levels, may have a distinct function.

A role for intronic m^6^A in regulation of splicing has been proposed previously in relation to female-specific *Sxl* splicing and sex determination in *Drosophila* (Haussmann et al. 2016; Lence et al. 2016). Consistent with this idea, we observe preferential association of intronic m^6^A with alternatively spliced regions and, moreover, following acute depletion of METTL3, widespread perturbation of splicing events in nascent RNA. Thus, we find that RNA m^6^A modifications are located in alternative introns/exons, including alternative 5′-splicing, alternative 3′-splicing, alternative last exons, and exon skipping isoforms, and that they correlate with inclusion of this alternative part as “exon” in the nascent transcriptome. Our observations support that high-confidence m^6^A methylation in constitutive introns is rare, as reported previously (Ke et al. 2017). Based on the assumption that m^6^A affects splice site usage in *cis*, we further found that the location of m^6^A peaks relative to the 5′SS correlates with the splicing output. This study suggests that m^6^A when deposited in proximity may repress 5′ splice sites for both A5SS and ALE and thereby promotes the alternative site located downstream, that is, d5′SS in A5SS or poly(A) site in ALE. Although we provide several lines of evidence in support of this conclusion, direct causation of splicing by splice-site proximal m^6^A in *cis* warrants further investigation.

Our analysis extends previous models that either only covered exon skipping (Xiao et al. 2016) or focused on intron retention (Fish et al. 2019). Although we observed some overlap with splicing changes seen in prior studies—for example, in the *Tor1aip2* gene (Fig. 5)—our analysis detected many more instances of m^6^A-mediated differential splicing. The fact that our observations followed acute depletion of METTL3 suggests that the changes are directly linked to METTL3 function rather than secondary long-term effects from perturbing the m^6^A system. A likely explanation for the relatively high number of aberrant splicing events that we detected is that we analyzed splicing patterns in nascent RNA rather than processed mRNA. It follows that the affected introns/exons are underrepresented in processed mRNA samples, either because they are highly unstable or because they escape export and are retained in the nuclear chromatin fraction. Consistent with the former possibility, recent work has shown that m^6^A marks RNA transcribed from specific repeat sequence elements for degradation by the nuclear exosome (Liu et al. 2020).

We find evidence that genes encoding several subunits of m^6^A writer and reader complexes have m^6^A-dependent splicing. Moreover, we observed that protein levels of some of these factors, such as YTHDC1 and WTAP, increase following acute depletion of METTL3, indicating that feedback mechanisms have evolved to regulate m^6^A-dependent functions. In these cases, the m^6^A-bearing alternative exon parts are “poisonous” and do not produce the full-length functional protein. This auto-regulation via unproductive splicing is reminiscent of that seen in the SR family of splicing regulators (Lareau et al. 2007). We infer that the m^6^A-dependent splice form suppresses levels of the major protein-coding splice variants, either as a result of altering ratios of translationally productive and nonproductive mRNA or by a function for the nonproductive transcript in transcription/translation in *cis* or in *trans*. It will be interesting in the future to further investigate this idea and to determine if other m^6^A-dependent splicing events play a role in regulating and/or fine-tuning different biological pathways. The use of acute METTL3 depletion, in allowing discrimination of direct and indirect deficits, will be an important tool for any such studies. Of note, our finding that acute depletion of METTL3 leads to degradation of METTL14, to which it is stably bound, but not of other accessory proteins such as WTAP and RBM15 provides support to the suggestion that METTL3/14 form a stable subcomplex distinct from the accessory proteins (Bokar et al. 1997; Knuckles et al. 2018).

In summary, we have defined the pattern of intronic m^6^A modification in the mESC nascent transcriptome and shown that intronic m^6^A mediates inclusion of alternative intron/exons, highlighting a potentially important level of gene regulation for the evolution and fine-tuning of biological pathways.

## Methods

### Cell lines

E14 mESCs were used for ChrMeRIP-seq. Hybrid (Cast/129S) XX mESCs containing inducible *Xist* on the Cast allele (Nesterova et al. 2019) were used to knock-in *FKBP12*^*F36V*^ into the *Mettl3* locus. The emGFP-PreScission-RBM15 cell line was derived from mouse XY 3E ESCs, containing rtTA integrated into the *Rosa26* locus and a random integration of the Dox-inducible *Xist* transgene into Chr 17 (Tang et al. 2010; Coker et al. 2020). In these cells, the puromycin resistance cassette at the *Rosa26* locus was replaced with hygromycin resistance (Moindrot et al. 2015). Then, cells were transfected and screened for stable integration of the pTRE-emGFP-PreScission-RBM15 plasmid. Cells treated with 1 μg/mL Dox for 24 h simultaneously induce *Xist* RNA and emGFP-PreScission-RBM15 protein expression. Primers and antibodies used to generate cell lines are listed in Supplemental Tables S3 and S4.

### ChrMeRIP-seq and calibrated MeRIP-seq

MeRIP-seq was based on the method by Dominissini et al. (2013) with minor modifications. Briefly, total RNA or ChrRNA was isolated from preplated mESCs according to the procedure above. RNA was fragmented by incubation for 6 min at 94°C in thin-walled PCR tubes with fragmentation buffer (100 mM Tris-HCl, 100 mM ZnCl_2_). Fragmentation was quenched using stop buffer (200 mM EDTA, pH 8.0) and incubation on ice, before ensuring the correct size (∼100 bp) using RNA Bioanalyzer. Total RNA isolated from *Drosophila* SG4 cells was also fragmented in parallel. For ChrMeRIP (conventional MeRIP-seq on chromatin-associated RNA), ∼50 μg ChrRNA was used. For calibrated MeRIP-seq using total RNA isolated from dTAG13-treated and untreated control cells (C3 and H5, depicted in Fig. 4), 300 μg of fragmented (∼100 nt) RNA, supplemented with 30 μg fragmented *Drosophila* total RNA, was mixed in m^6^A IP buffer. RNAs were incubated with 10 μg anti-m^6^A antibody (Synaptic Systems, 202 003; or Abcam #ab151230), RNasin (Promega), 2 mM VRC, 50 mM Tris, 750 mM NaCl, and 5% IGEPAL CA-630 in DNA/RNA low-bind tubes for 2 h before m^6^A-containing RNA was isolated using 200 μL Protein A magnetic beads per IP (preblocked with BSA). After this 2-h incubation, extensive washing (1× IP buffer [10 mM Tris-HCl, pH 7.4, 150 mM NaCl, 0.1% NP-40], 2× LowSalt buffer [50 mM Tris-HCl, pH 7.4, 50 mM NaCl, 1 mM EDTA, 1% NP-40, 0.1% SDS], 2× HighSalt buffer [50 mM Tris-HCl, pH 7.4, 1 M NaCl, 1 mM EDTA, 1% NP-40, 0.1% SDS], 1× IP buffer) was performed to remove the unspecific binding. To elute RNA from the beads, 6.7 mM m^6^A (Sigma-Aldrich) was used. Input and eluate samples were EtOH-coprecipitated with Glycoblue, quantified, and pooled as libraries generated using TruSeq Stranded total RNA LT Sample Prep (Abcam ChrMeRIP-seq experiment) or NEBNext Ultra II Directional RNA Library Prep (SySy ChrMeRIP-seq) according to the manufacturer's instructions but skipping the fragmentation step. Seventy-five-base pair single-end reads were obtained using Illumina NextSeq 500.

### RNA m^6^A modification peak calling and confidence group classification

We performed peak calling on duplicate m^6^A IP and input alignment BAM files with the MACS2 (v2.1.1) tool (Zhang et al. 2008). SySy and Abcam ChrMeRIP-seq data were analyzed separately. Nascent transcriptome size was calculated from the UCSC Genome Browser and used as genome size. The key parameters were (*-q 0.05 ‐‐nomodel ‐‐extsize 100 ‐‐call-summits*) in addition to the genome size (*gsize*) 2.4E8 for ChrMeRIP and 1.05E8 for standard MeRIP-seq (Dominissini et al. 2013). The strand-specific bigWig files were generated from RNA-seq by BEDTools (Quinlan and Hall 2010), and peak strands were determined by calculating the strand-specific fold change log_2_(IP/Input) using the UCSC utility *bigWigAverageOverBed*. Direction-ambiguous peaks were removed from the analysis. The borders of m^6^A peaks were further refined according to the “summits” output from MACS2. Given that RNAs were fragmented to a size slightly longer than 100 nt for MeRIP-seq, we shrunk the peak size by only keeping the 125 nt on each side of the summit if the called m^6^A peak was larger than 250 nt. Broad peaks were separated into individual subpeaks if multiple summits were called by MACS2. If summits were separated by less than 250 nt, the boundary between peaks was set at the middle site between summits. The custom scripts used for these refinements are included as Supplemental Code. This analysis resulted in 10,749 and 11,726 peaks for SySy and Abcam antibody, respectively. We further defined the high-confidence m^6^A set (ConfGroup1, *n* = 5277) as peaks called from both antibodies, the medium-confidence m^6^A set (ConfGroup2, *n* = 5472) as peaks detected by the SySy antibody and also having signal from the Abcam antibody but below the cutoff to be called peaks by MACS2 (Zhang et al. 2008), and the low-confidence m^6^A set (ConfGroup3, *n* = 6319) as peaks detected only by the Abcam antibody and almost no signal from the SySy antibody. Motifs for each group were searched and analyzed by the HOMER program (Heinz et al. 2010).

### RNA m^6^A peak intensity analysis

MeRIP-seq reads were split into positive and negative strands (Li et al. 2009), and bigWig files were generated accordingly. Ten million mapped reads per library were used to perform normalization in ChrMeRIP and conventional MeRIP-seq data, except for the calibrated MeRIP-seq analysis that was normalized to the sequenced *Drosophila* RNA reads (Supplemental Table S1). m^6^A peak intensity was calculated as log_2_(IP/Input), with peak intensity close to 0 or less than 0 indicating no m^6^A enrichment. m^6^A peaks were further classified as either peaks close to transcript start sites as determined by deep-sequenced nuclear CAGE libraries from E14 mESCs (GSE148382) (Wei et al. 2020), or peaks distal to TSS (intragenic and intergenic m6A peaks). Intragenic m^6^A peaks were further grouped into intronic peaks and exonic peaks. Peaks closer to TSSs may be potential m^6^Am peaks because the m^6^A antibody cannot distinguish m^6^A and m^6^Am.

### RNAmpp analysis

RNA metaprofile plot scripts were written for this study. Step1: isoform selection (RNAmpp_prep.sh). Gene annotations were downloaded from GENCODE or UCSC Genome Browser as GTF format. Representative isoforms for each gene can be chosen in three ways: (1) one isoform at random; (2) by the “MaxORF_LongestNcRNA” method, which for protein-coding genes chooses the transcript with the maximal open reading frame (ORF), or the longest transcript for lncRNAs or if multiples isoforms have equal maximum ORFs; or (3) the user-defined custom isoform. Step2: relative position calculation (RNAmpp_stat.sh). First, the single strand-specific single nucleotide position (Bed-format) called from m^6^A-seq (peak summits) or irCLIP-seq (CITS) is used as the input to find the intersecting gene in a strand-specific manner with intersectBed (-s) from BEDTools (Quinlan and Hall 2010), then the introns or exons of overlapping genes are iterated to calculate the relative position of the query site to the gene feature (for protein-coding genes, this is relative to the start position of the particular feature, i.e., 5′ UTRs, CDSs, 3′ UTRs, for lncRNAs relative to the TSS, and for introns relative to the 5′ splice site). Given that 5′ UTRs, CDSs, and 3′ UTRs have variable lengths in different genes, the average length for all 5′ UTRs (221.23 nt), CDSs (1638.32 nt), and 3′ UTRs (1252.16 nt) are calculated from GENCODE vM24 annotation. These calculations are used to determine the number of bins to use for each type of feature so that each bin is, on average, the same sequence length, allowing for direct comparison among different mRNA regions. For introns, each intron is split into 40 bins with equal size. Pie charts and metaprofiles are generated by R packages (R Core Team 2019). The dashed lines in metaprofiles denote the CDS start and CDS stop. Pie charts have two levels: level 1 shows exon, intron, and intergenic fractions, whereas level 2 further shows the protein coding fraction within exons and introns. RNAmpp, implemented in Python 3 and R (v3.6) (R Core Team 2019) with dependency, is publicly available in GitHub.

### Alternative splicing analysis

4sU-seq data sets were mapped to the mm10 genome by STAR (v2.5.2b) (Dobin et al. 2013) with the following key parameters: (‐‐*twopassMode Basic ‐‐outSAMstrandField intronMotif ‐‐outSAMattributes All ‐‐outFilterMultimapNmax 1 ‐‐outFilterMismatchNoverReadLmax 0.06 ‐‐alignEndsType EndToEnd*). For intron-centric differential splicing analysis, the LeafCutter package (v0.2.7) (Li et al. 2018) was used to quantify intron usage and identify differentially spliced intron clusters between two conditions (dTAG-13 treated and nontreated control, or wild type vs. *Mettl3* knockout). Only splice junctions supported by uniquely mapped reads were used. Our analysis followed the differential splicing documents from the package, except the split reads number for intron cluster. For the dTAG METTL3 experiment, at least 30 split reads (six replicates) are required but 25 for the published *Mettl3* knockout data sets (GSE86336 from Ke et al. 2017, GSE61997 from Geula et al. 2015). The splicing clusters were ranked, and three different thresholds were set to generate differential splicing levels (for the dTAG METTL3 experiment, q < 0.05, q < 0.2, p < 0.05 were chosen; for the *Mettl3* knockout data sets, q < 0.01, q < 0.05, p < 0.05 were chosen; here, q is the adjusted *P*-value), considering that the RNA spliced reads coverages are different. If the splicing cluster has one (or multiple) m^6^A peak(s) from any confidence group overlapped or within 500 bp distance, it was considered as a *cis*-m^6^A-regulated cluster, otherwise a “nom^6^A” cluster. Sashimi plots with deltaPSI were generated from the LeafCutter package (Li et al. 2018). Peak intensity from each m^6^A peak was calculated as above, and the average m^6^A peak number was calculated from each cluster. The splicing choice score for alternative 5′ splice sites was defined by splicing reads covering the short splicing junction (d5′SS) divided by the total splicing reads covering both splicing junctions in this intron.

Three splicing types are described in this study based on the intron-centric analysis from LeafCutter output, which is slightly different from the actual classification which has A5SS, A3SS, IR, ES, MXE, AFE, and ALE types. Here, we described three splicing types: SAS, ES, and pf_IR (Fig. 5). SAS (splicing alternative site) consists of two or more alternative splicing sites in the cluster that share either the start or the end coordinate. This type mostly covers the alternative 5′ splice site and alternative 3′ splice site (A5SS or A3SS), as well as alternative first or last exon (AFE or ALE) in some cases. ES is the same as the exon skipping model, where a cassette exon is either included or excluded in the spliced product. Partial or full intron retention refers to where the splicing junction partially or fully locates inside of any exon (could be the first or last exon), indicating that the partial or full intron is included in the splicing product. This splicing type analysis was only focused on the clusters showing significant splicing changes. To avoid batch effects, we did the comparisons for the same splicing types from the same experiment with and without m^6^A modifications in the alternative exon/intron part (Fig. 5). The distance between the 5′ splice site and m^6^A peak summit was calculated using BEDTools (Quinlan and Hall 2010) (*closestBed -a 5′-splice_site.bed -b m6A_peak_summit.bed -s -D a*).

## Data access

High-throughput sequencing data (ChrMeRIP-seq, RBM15 irCLIP, calibrated MeRIP-seq, and 4sU-seq) generated in this study have been submitted to the NCBI Gene Expression Omnibus (GEO; https://www.ncbi.nlm.nih.gov/geo/) under accession number GSE154709. The UCSC Genome Browser view of the ChrMeRIP-seq, RBM15 irCLIP-seq, and CAGE-seq (GSE148382) can also be accessed (http://genome-euro.ucsc.edu/s/Guifeng/ChrMeRIP). Scripts (https://github.com/guifengwei/Nascent_m6A_Scripts) and RNAmpp analysis (https://github.com/guifengwei/RNAmpp) in this study can be found as Supplemental Code. The plasmids used for generation of METTL3-FKBP12^F36V^ have been deposited in Addgene under 165420 (pSpCas9_U6_sgRNA_Mettl3C) and 165421 (pTargeting_Mettl3C_FKBP-V).

## Supplementary Material

Supplemental Material
