# Supplementary material for: Acute depletion of METTL3 implicates N6-methyladenosine in alternative intron/exon inclusion in the nascent transcriptome
Source: Genome Res. 2021 Aug;31(8):1395–408. doi: 10.1101/gr.271635.120 (PMC8327914; doi:10.1101/gr.271635.120)
Supplement: Supplemental Material [file supp_31_8_1395__DC1.html]

Acute depletion of METTL3 implicates N6-methyladenosine in alternative intron/exon inclusion in the nascent transcriptome — Supplemental Material 

# Acute depletion of METTL3 implicates *N*6-methyladenosine in alternative intron/exon inclusion in the nascent transcriptome

## Supplemental Material

- Supplemental\_Data\_S1.zip
- Supplemental\_Data\_S2.zip
- Supplemental\_Data\_S3.zip
- Supplemental\_Code.tar.gz.zip
- Supplemental\_materials\_v11.1.pdf
