# Supplementary figures and images for: Acute depletion of METTL3 implicates N6-methyladenosine in alternative intron/exon inclusion in the nascent transcriptome
Source: Genome Res. 2021 Aug;31(8):1395–408. doi: 10.1101/gr.271635.120 (PMC8327914; doi:10.1101/gr.271635.120)

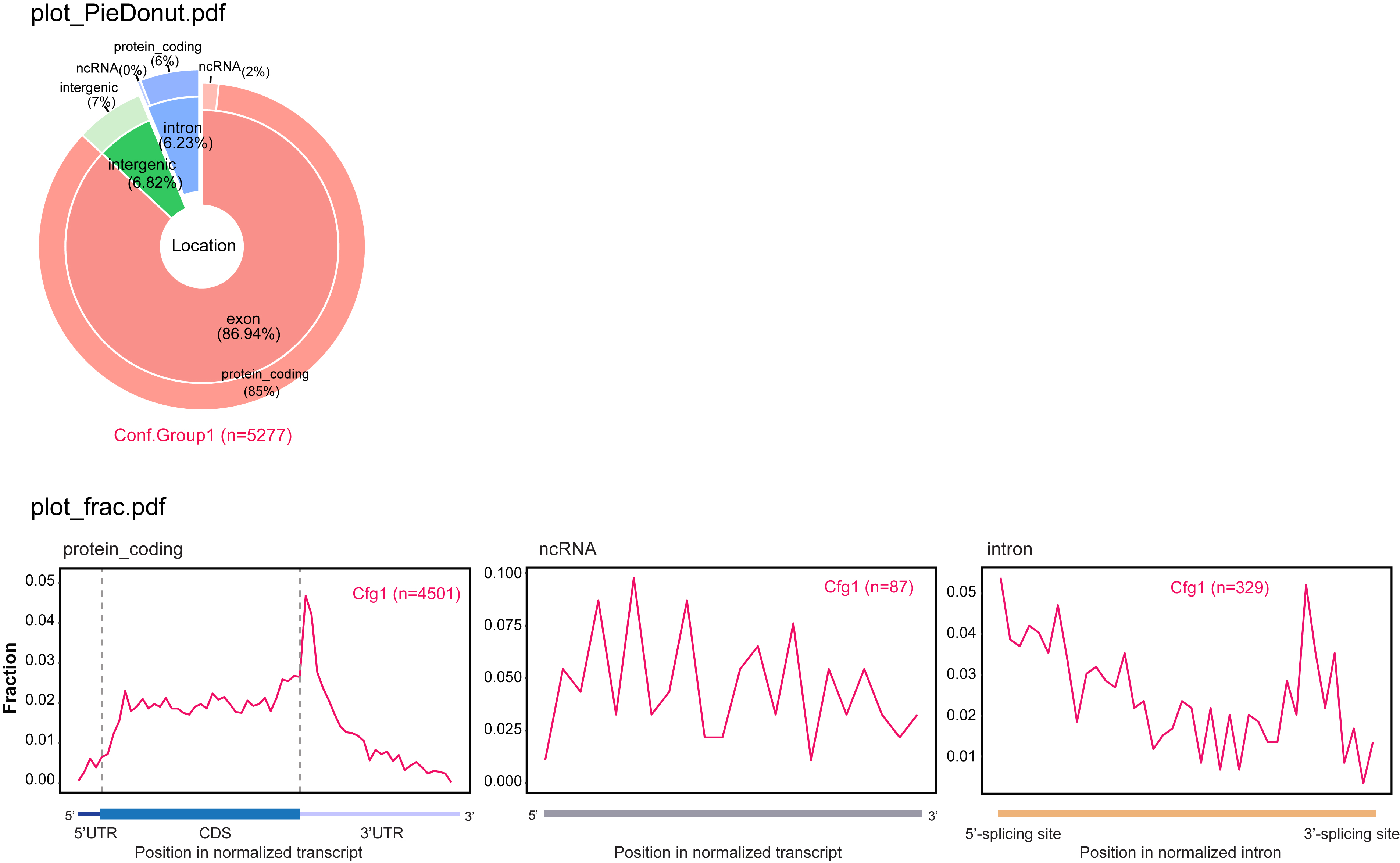

Supplement: Supplemental Material [file supp_gr.271635.120_Supplemental_Code.tar.gz.zip › RNAmpp/RNAmpp.jpg]

**Histogram of data\$V1**

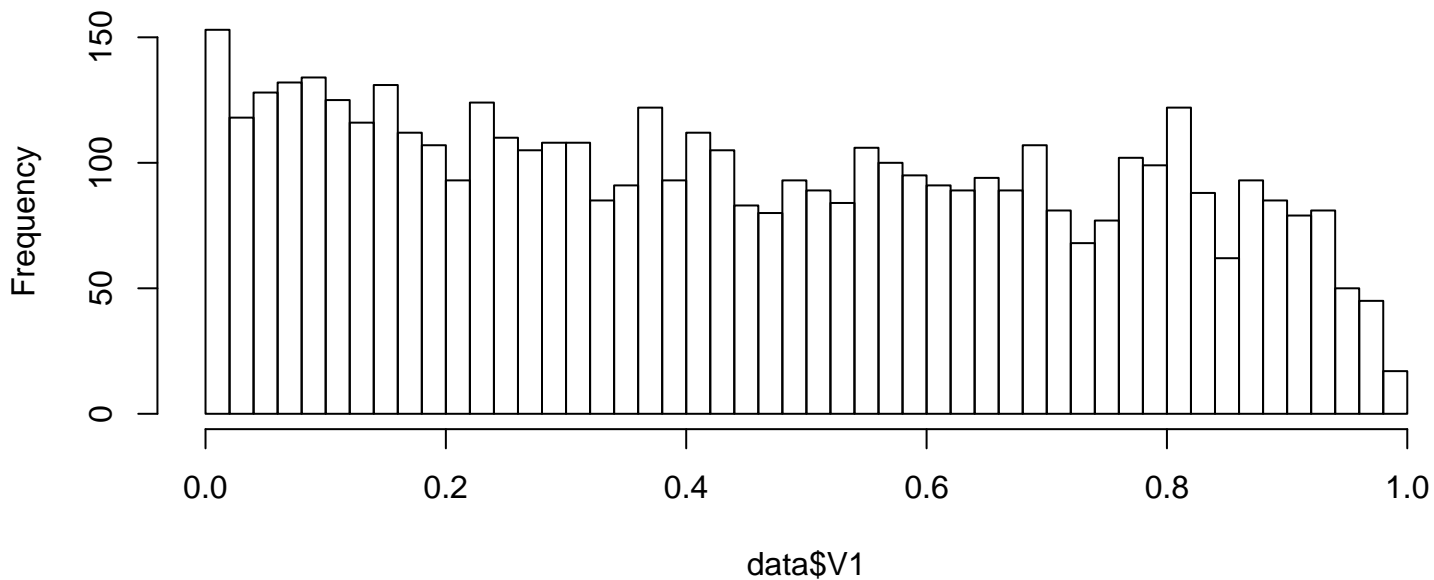

Supplement: Supplemental Material [file supp_gr.271635.120_Supplemental_Code.tar.gz.zip › Nascent_m6A_Scripts/intron_methylation/Intron2TSS.pdf]
