# Supplementary material for: Acute depletion of METTL3 implicates N6-methyladenosine in alternative intron/exon inclusion in the nascent transcriptome
Source: Genome Res. 2021 Aug;31(8):1395–408. doi: 10.1101/gr.271635.120 (PMC8327914; doi:10.1101/gr.271635.120)
Supplement: Supplemental Material [file supp_gr.271635.120_Supplemental_materials_v11.1.pdf]

Supplemental Materials for

Acute depletion of METTL3 implicates N^6-methyladenosine in alternative intron/exon inclusion in the nascent transcriptome

Guifeng Wei *et al.*

Department of Biochemistry, University of Oxford, Oxford, UK

Contents

Supplemental Figures S1-S15.

Supplemental Methods.

Supplemental Table S1: Summary of ChrMeRIP-seq data.

Supplemental Table S2: Calibrated MeRIP sequencing depth for normalization.

Supplemental Table S3: Primers used in this study.

Supplemental Table S4: Antibodies used in this study.

Other Supplementary Materials for this manuscript

Supplemental Data S1: Confidence m^{6}A peaks characterized in this study.

Supplemental Data S2: Differentially expressed genes between control and dTAG-13 treatment.

Supplemental Data S3: Splicing changes calculated from LeafCutter.

Supplemental_Fig_S1

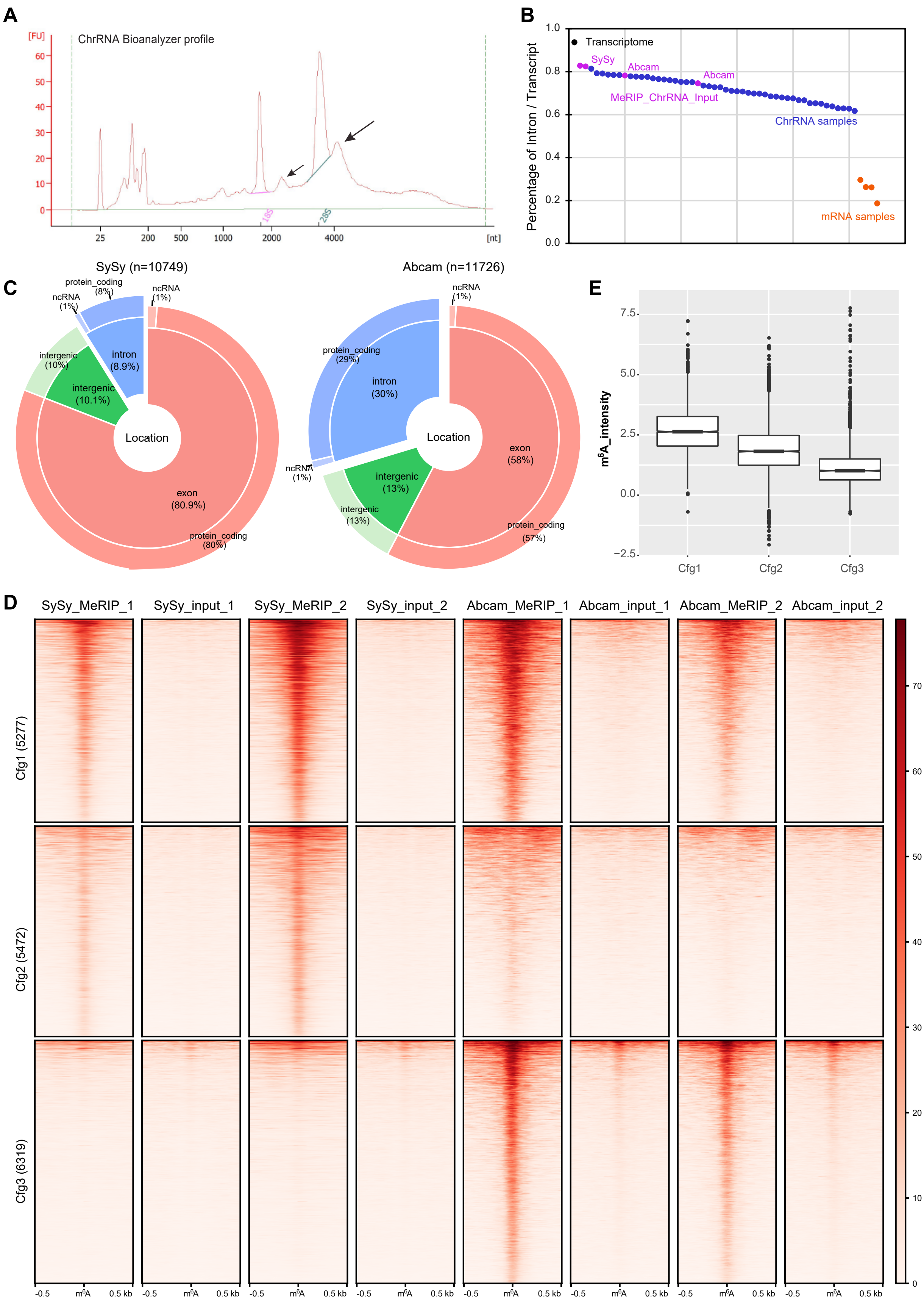

Supplemental Figure S1. Characterization of the nascent m^{6}A transcriptome by ChrMeRIP-seq, related to Figure 1.

(A) RNA Bioanalyzer traces of ChrRNA samples. Arrowheads indicate the two subpeaks after 18S and 28S rRNA representing pre-rRNA.

(B) The intronic percentage of ChrMeRIP-seq samples (purple) lined up with ChrRNA-seq samples from our previous study (Nesterova et al. 2019). The intronic proportion of the annotated transcriptome (mm10) and mRNA samples (orange) are displayed for comparison.

(C) Pie chart comparisons of the distribution of m^{6}A summits called using the SySy antibody (left) and Abcam antibody (right).

(D) Heatmap showing the signal for called m^{6}A peaks in each replicate of m^{6}A IP and input samples for all the confidence group. The centre of m^{6}A peak stands for the peak summit called by MACS2 and the flanking strand-specific 500nt regions were taken for calculating the signal and making plot. The color key is shown aside.

(E) Boxplots showing the distribution of m^{6}A peak intensities for each confidence group, calculated as averages over 4 replicates (2 SySy + 2 Abcam).

Supplemental_Fig_S2

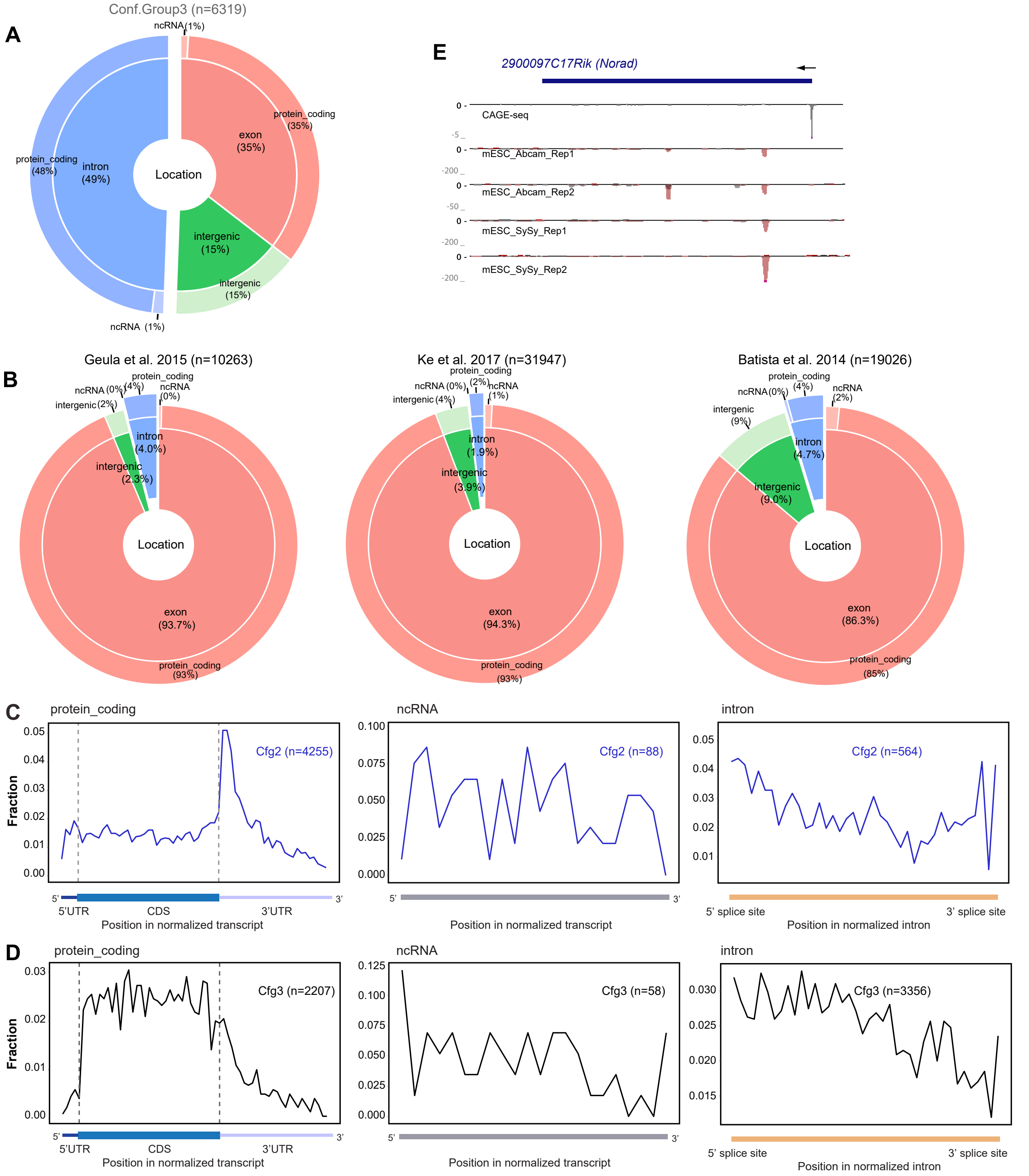

Supplemental Figure S2. Comparison of m^{6}A peaks from ChrMeRIP-seq and standard MeRIP-seq, related to Figure 1.

(A) Pie charts showing the distribution of m^{6}A peaks for Cfg3 group. Same as Fig. 1D.

(B) Pie charts showing m^{6}A peak distributions from two previous studies with MeRIP (Batista et al. 2014; Geula et al. 2015) and one study with m^{6}A-CLIP in mESCs (Ke et al. 2017).

(C,D) Same as Fig. 1F but for Cfg2 (C) and Cfg3 (D) groups.

(E) UCSC Genome Browser screenshot showing an example lncRNA (*Norad*) harboring m^{6}A methylation. From top to bottom, tracks denote gene annotation, CAGE-seq and ChrMeRIP-seq (Abcam 2 replicates, SySy 2 replicates).

Supplemental_Fig_S3

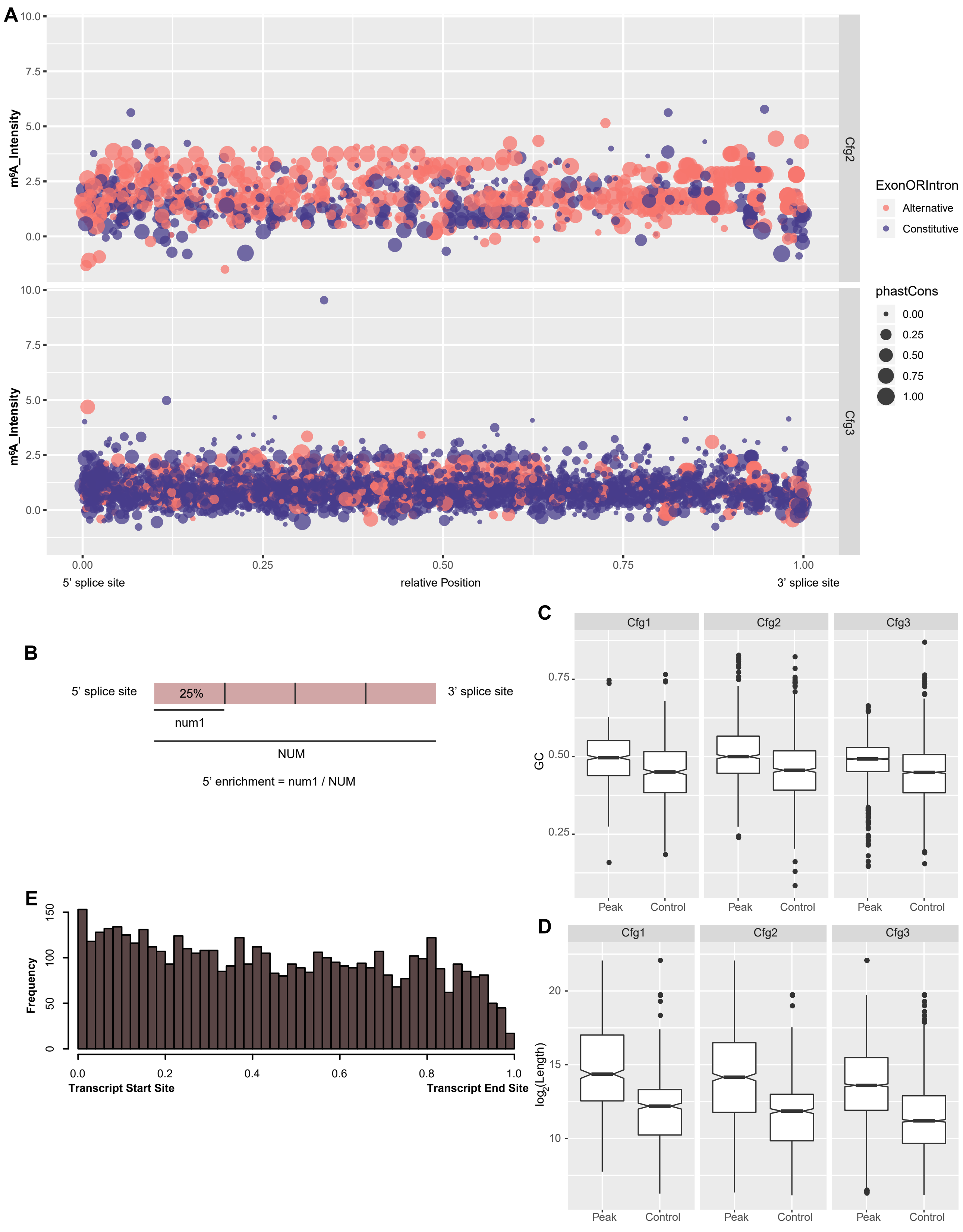

Supplemental Figure S3. Intronic m^6A methylation patterns, related to Figure 2.

(A) Same as Fig. 2A but for all intronic m^6A peaks from Cfg2 and Cfg3 groups.

(B) Schematic showing methodology used to calculate enrichment in the most 5' 25% of introns, used in Fig. 2B.

(C) Boxplots showing GC percentage of intronic m^6A peaks compared to matched controls for all confidence groups.

(D) Same as (C) but for length of introns hosting m^6A peaks. Controls are gene matched.

(E) Histogram showing intronic m^6A peaks relative to annotated transcription start sites, using representative isoforms from MaxORF_LongestNcRNA.

Supplemental_Fig_S4

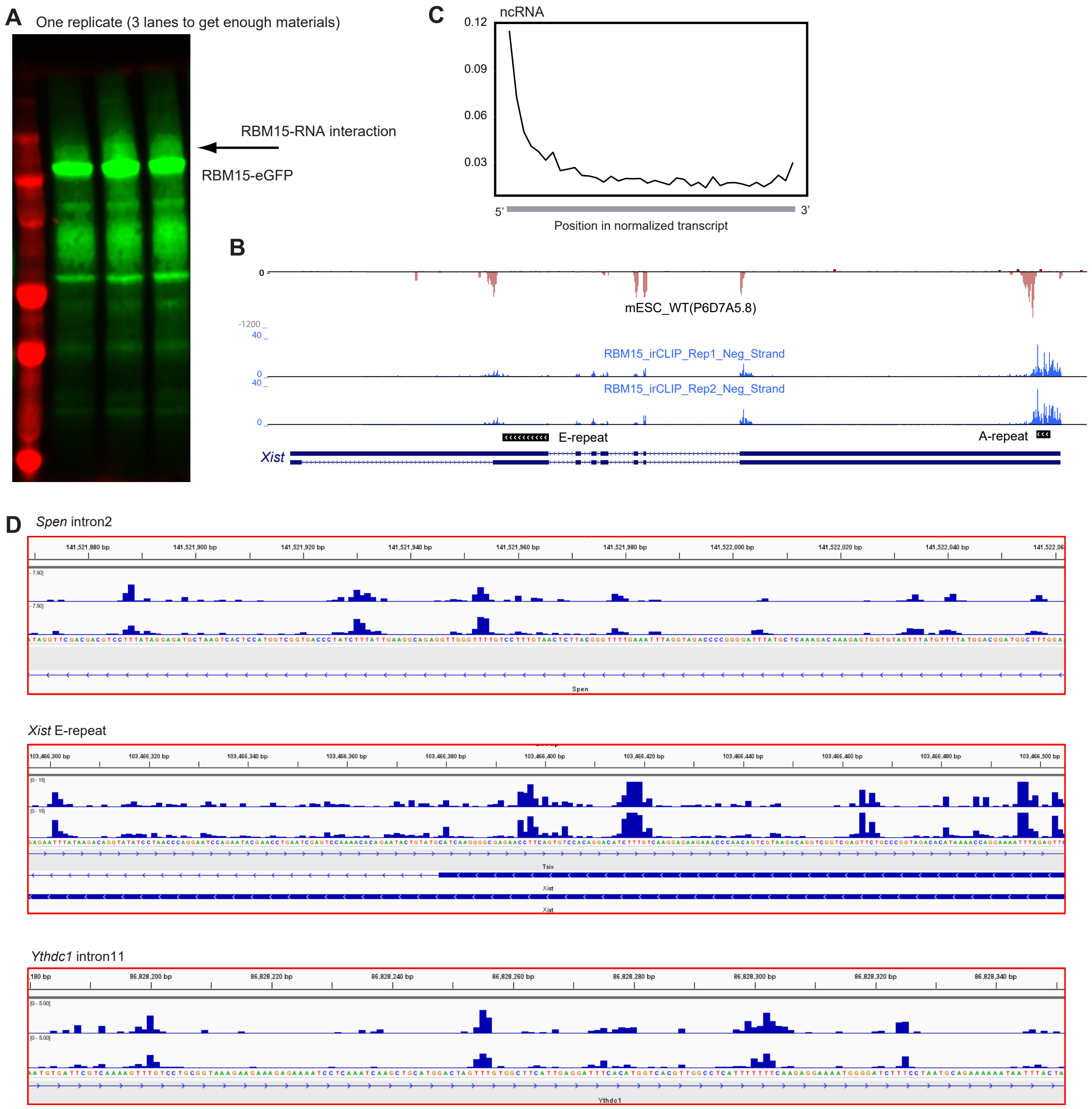

Supplemental Figure S4. Performance of RBM15 irCLIP-seq, related to Figure 3.

(A) Odyssey image showing successful transfer of UV crosslinked RNA-protein. The arrowhead indicates the shift caused by crosslinking of emGFP-RBM15 with its interacting RNAs. Materials from all 3 lanes were used for one replicate irCLIP-seq experiment.

(B) UCSC genome browser tracks showing the profile of m^6A in *Xist* RNA, aligned with the two replicates of RBM15 binding. RT stops (i.e. the 5'-end of each sequencing read) were shown for the irCLIP-seq tracks. Landmark A- and E-repeats of *Xist* are also indicated.

(C) Metaprofile of RBM15 binding (CITS>=3 in two replicates) over noncoding RNA, calculated by RNAmpp analysis.

(D) Zoomed-in view of strand-specific RBM15 binding on loci of example genes: *Spen* intron 2 (top), *Xist* E-repeat (middle), and *Ythdc1* intron 11 (bottom). RT stops are shown in the tracks. Direction of RNA transcription is also indicated.

Supplemental_Fig_S5

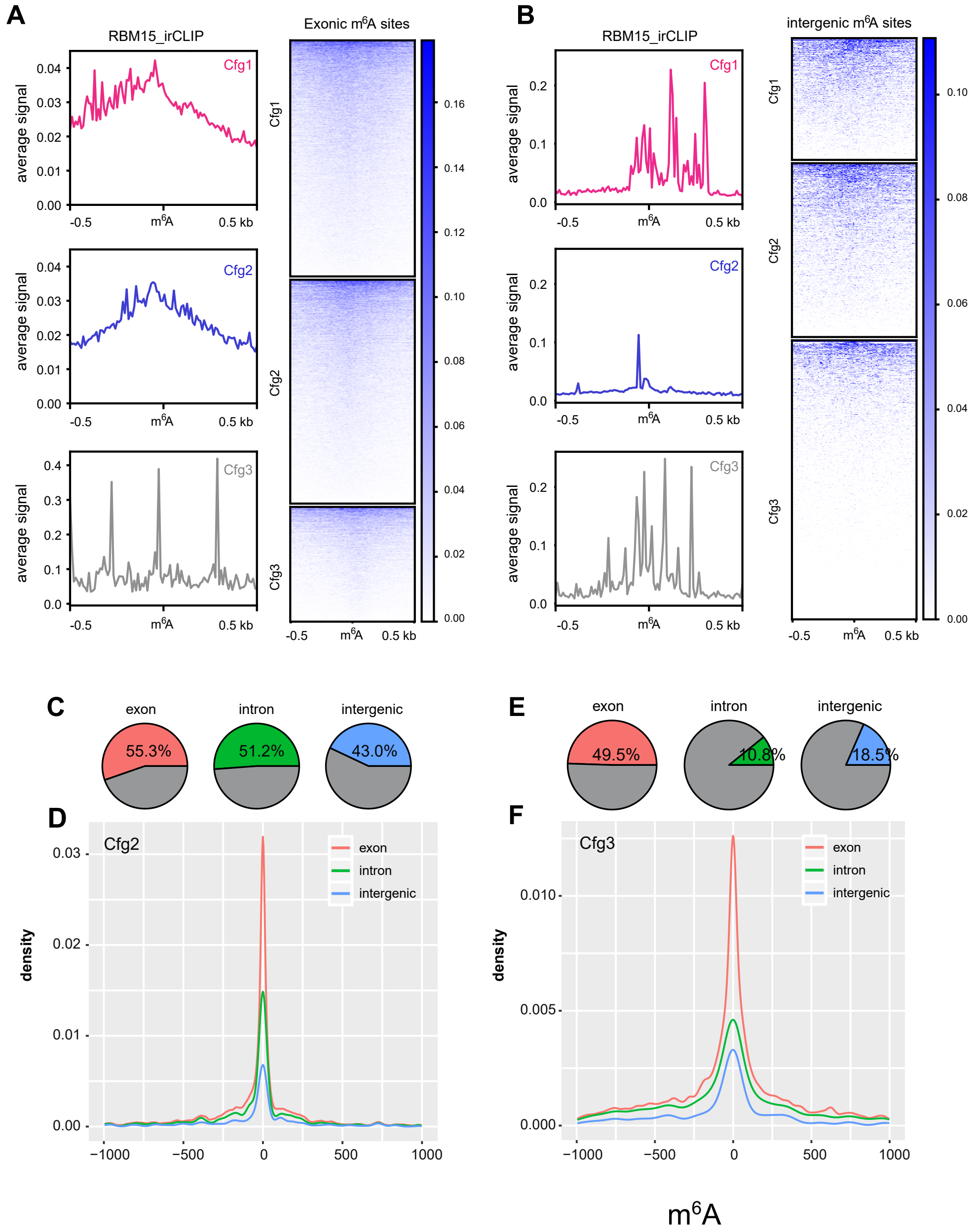

Supplemental Figure S5. RBM15 binding correlates with m^{6}A modification, related to Figure 3.

(A) RBM15 binding (CITS) metaprofiles and heatmaps for exonic m^{6}A peaks of 3 different confidence groups (red, blue, and grey for Cfg1, Cfg2, and Cfg3 respectively). The color key is shown for all heatmaps. 0.5kb strand-specific flanking regions each side of m^{6}A peak summit are included for the plot.

(B) Same as (A) but for intergenic m^{6}A peaks.

(C-F) Same as Fig. 3E,F but for Cfg2 groups (C,D) and Cfg3 groups (E-F).

Supplemental_Fig_S6

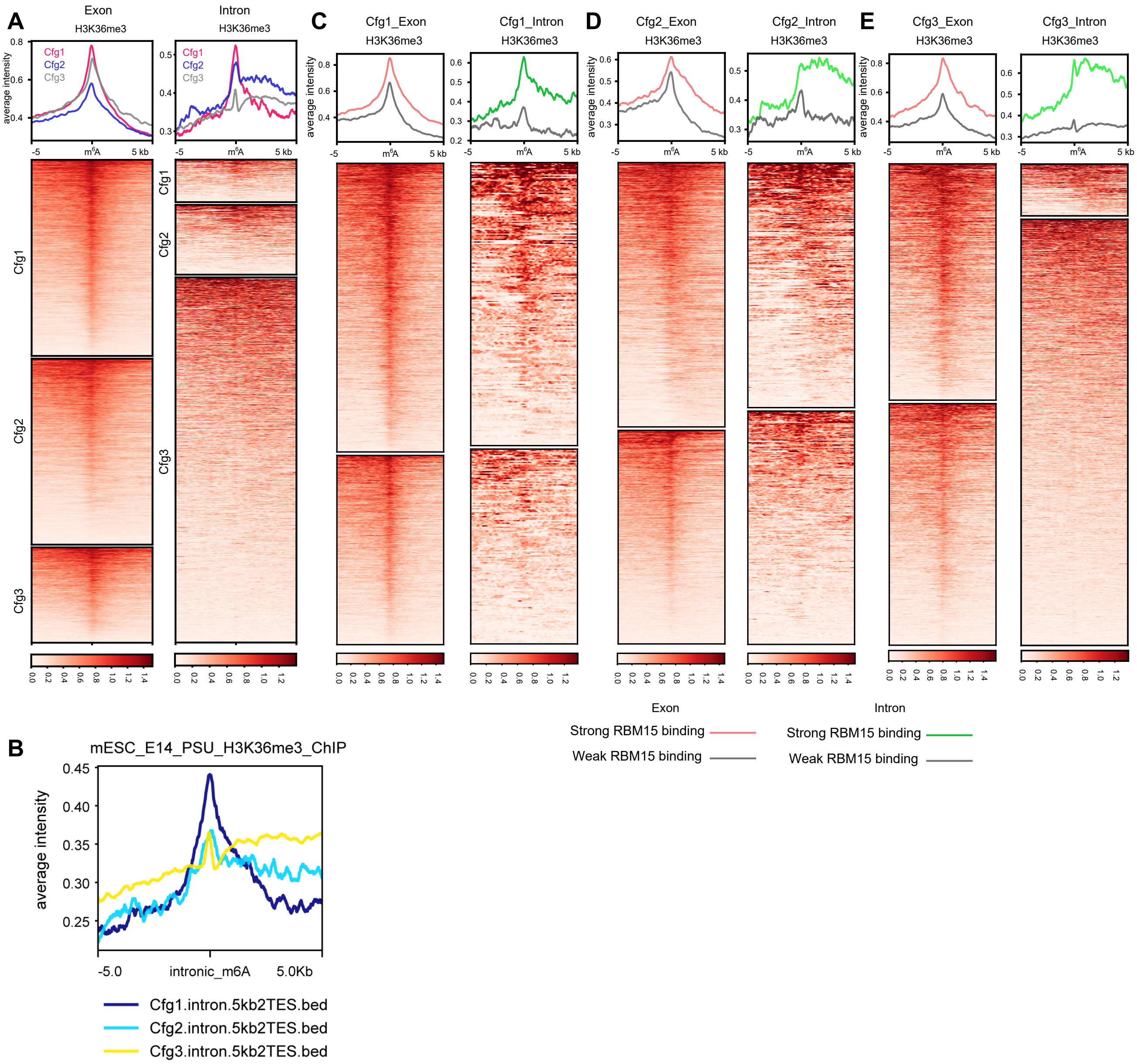

Supplemental Figure S6. H3K36me3 correlates with RBM15 binding and RNA m^{6}A modification, related to Figure 3.

(A) H3K36me3 metaprofiles and heatmaps for m^{6}A peaks from confidence groups Cfg1 (red), Cfg2 (blue) and Cfg3 (grey), ordered vertically (from top to bottom are Cfg1 to Cfg3) in the heatmaps. Exonic (left) and intronic m^{6}A peaks (right) are separated. Color keys are shown as bellow. 5kb flanking regions each side of the m^{6}A peak summit are included for the plot.

(B) Metagene profile of H3K36me3 at the intronic m^{6}A sites as well as flanking 5 kb regions. The intronic m^{6}A sites are more than 5 kb away toward the annotated transcription terminal site of MaxORF_LongestNcRNA transcript by comprehensive GENCODE_vM24 annotation.

(C-E) Metaprofiles and heatmaps of H3K36me3 for Cfg1 (C), Cfg2 (D), and Cfg3 (E) m^{6}A peaks with strong (red and green) and weak (grey) RBM15 binding. Red and green curves denote exonic and intronic m^{6}A peaks respectively. 5kb regions flanking each m^{6}A peak summit are included for the heatmaps, with color keys of H3K36me3 shown below.

Supplemental_Fig_S7

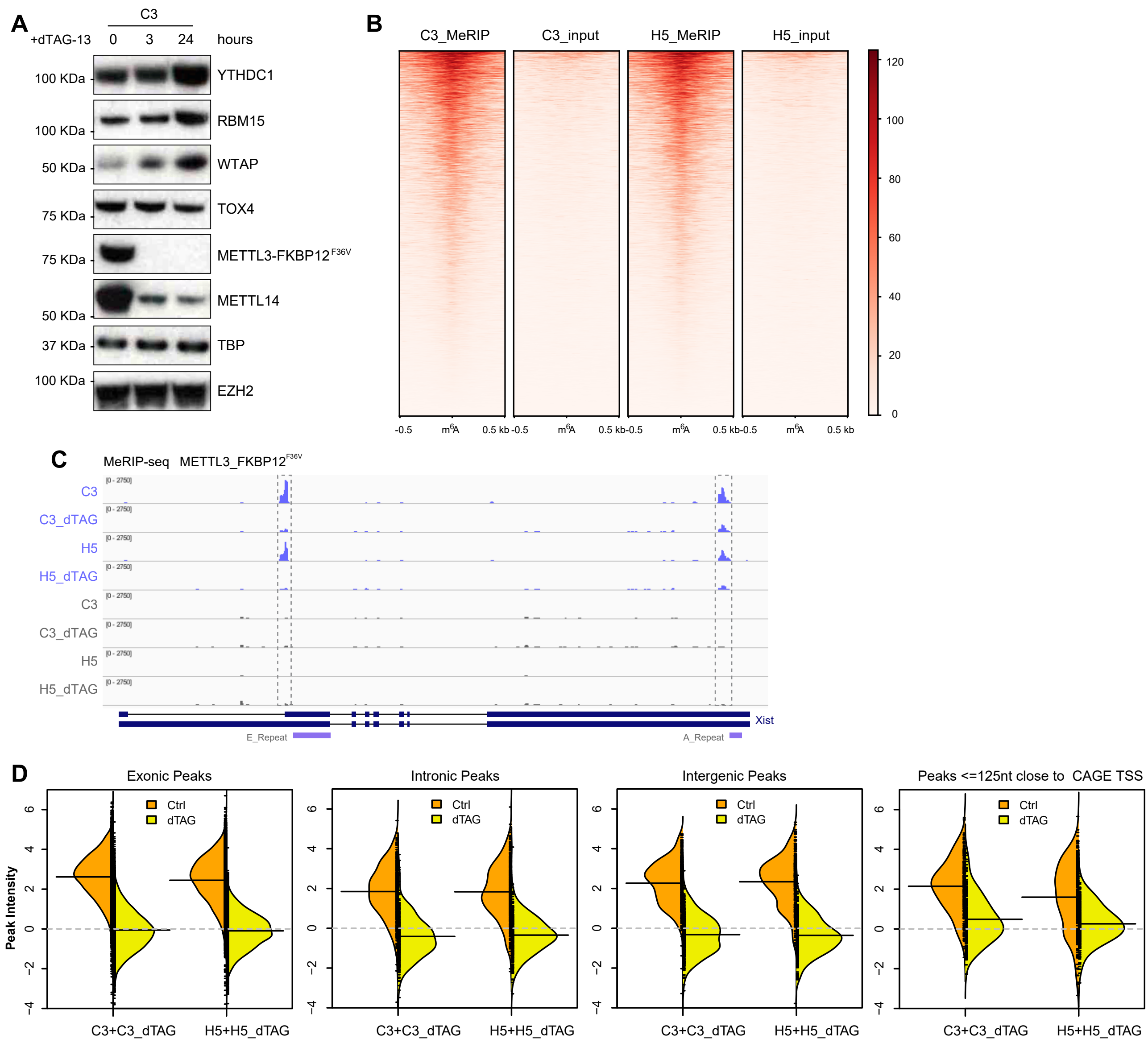

Supplemental Figure S7. Validation of functional m^{6}A loss by dTAG METTL3 in mESC, related to Figure 4.

(A) Additional replicate of western blot analysis shown in Fig. 4C.

(B) Heatmap showing the m^{6}A peak signals from two METTL3_FKBP12^{F36V} lines, C3 and H5. Both MeRIP and input samples are shown. The centre of m^{6}A peak stands for the peak summit called from SySy ChrMeRIP-seq by MACS2 and the flanking strand-specific 500nt regions were taken for calculating the signal and making plot. The color key is shown on the right.

(C) Genome browser tracks showing methylation changes on *Xist* RNA upon dTAG-13 treatment. Samples names are indicated. The two dominant m^{6}A peaks on *Xist* RNA lie proximally downstream of *Xist* A- and E-repeat regions.

(D) Same as Fig. 4E but using the conventional method of calculating peak intensity.

Supplemental_Fig_S8

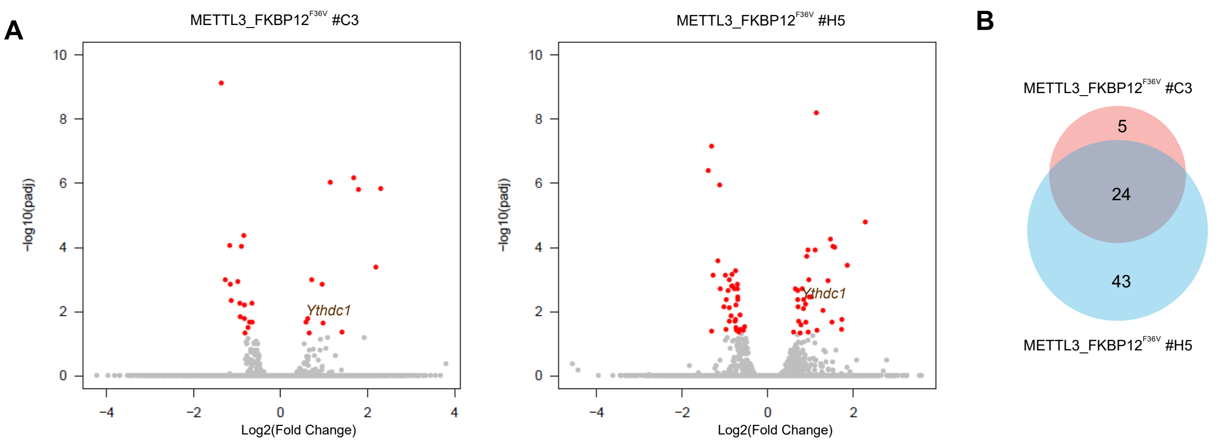

Supplemental Figure S8. Differentially expressed genes upon 3.5 hours of dTAG-13 treatment from 4sU-seq, related to Figure 5.

(A) Volcano plots show the differentially expressed genes (red dots) between control and dTAG-13 treatment samples from 3 independent replicates of METTL3_FKBP12^{F36V} clone C3 (left) and METTL3_FKBP12^{F36V} H5 clone H5 (right).

(B) Overlap of differentially expressed genes observed in the two clones.

Supplemental_Fig_S9

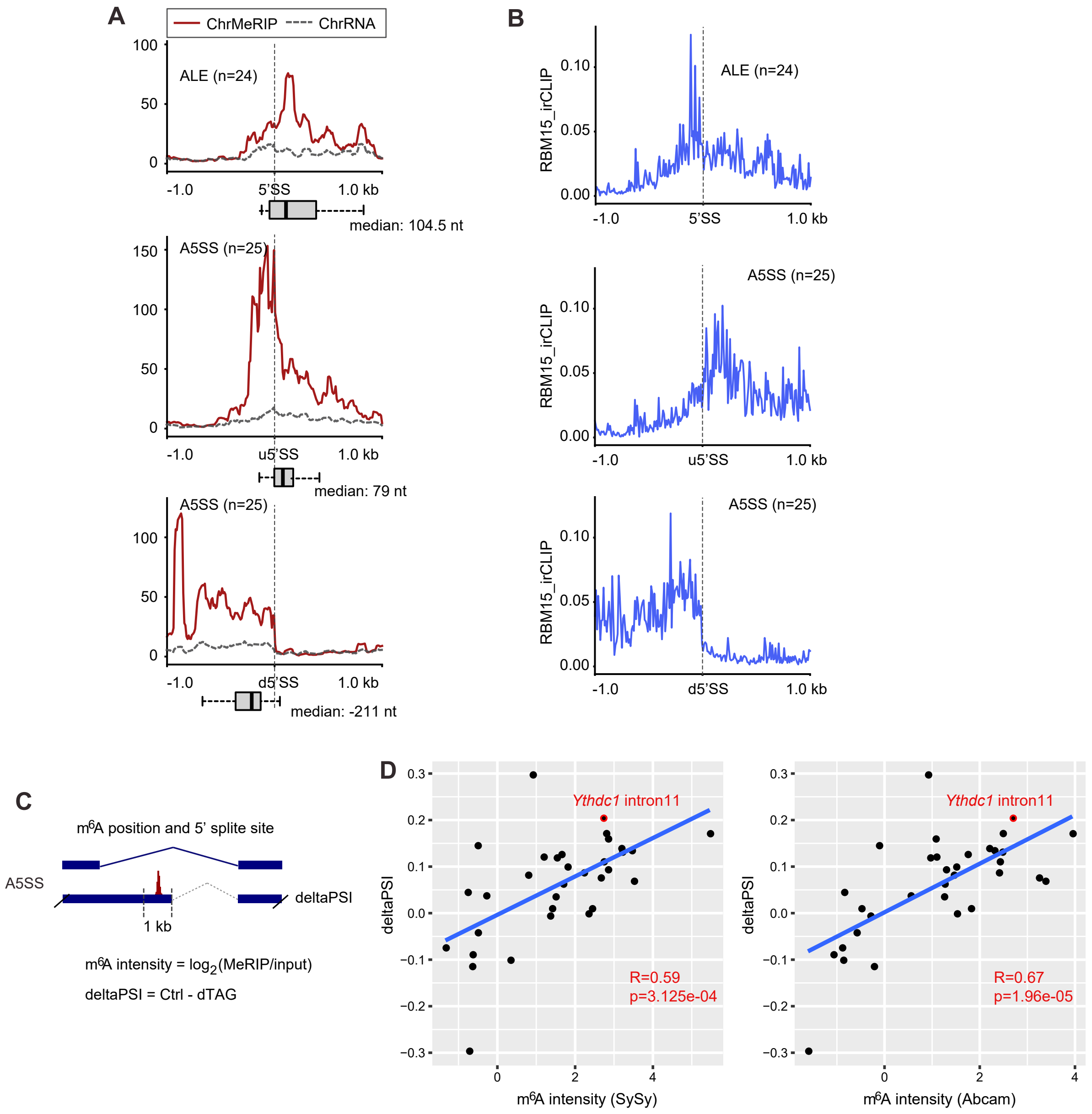

Supplemental Figure S9. Profile of m^{6}A and RBM15 binding toward splicing changed 5' splice sites from A5SS and ALE, related to Figure 5 and 6.

(A) Same as Fig. 6B-D but for Abcam ChrMeRIP-seq.

(B) Same as Fig. 6B-D but for RBM15 binding. Two irCLIP-seq replicates are averaged (n=24 for ALE and n=25 for A5SS).

(C,D) Schematic (C) and scatter plot (D) showing the correlation between m^{6}A intensity and the effect size of splicing changes (deltaPSI) calculated from dTAG METTL3 4sU-seq experiment. The intensity was calculated from 1000nt upstream of 5'SS (left: SySy 2 replicates; right: Abcam 2 replicates). Fitted lines (blue) and correlation coefficients are indicated. The dot denoting *Ythdc1* intron11 is also marked by red.

Supplemental_Fig_S10

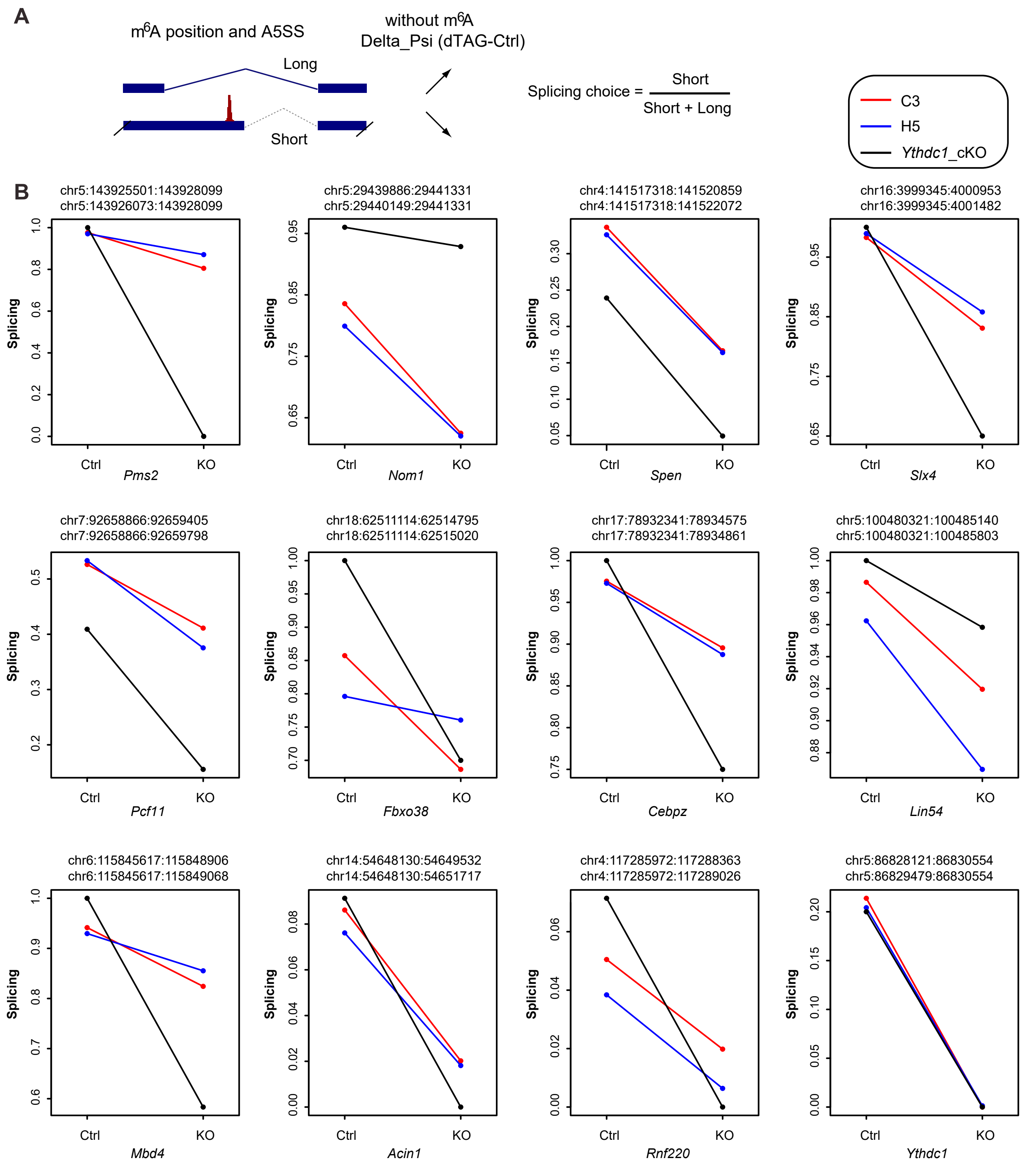

Supplemental Figure S10. Splicing choice score of short splicing form in m^{6}A-bearing alternative 5' splice sites, related to Figure 5 and 6.

(A) Schematic showing m^{6}A-bearing alternative 5' splice site. The splicing choice score is calculated as the proportion of short splicing form over all the splicing read numbers in this intron.

(B) The splicing direction for the m^{6}A-bearing short form upon acute depletion of METTL3 (red and blue lines denote METTL3_FKBP12^{F36V} clone C3 and H5 respectively from this study) or conditional *Ythdc1* knockout (black line, GSE133585). "Ctrl" indicates no treatment in the corresponding experiments, while "KO" means treatment for knockout. Coordinates of alternative 5' ss are shown above each panel and gene names are labelled at the bottom of each panel.

Supplemental_Fig_S11

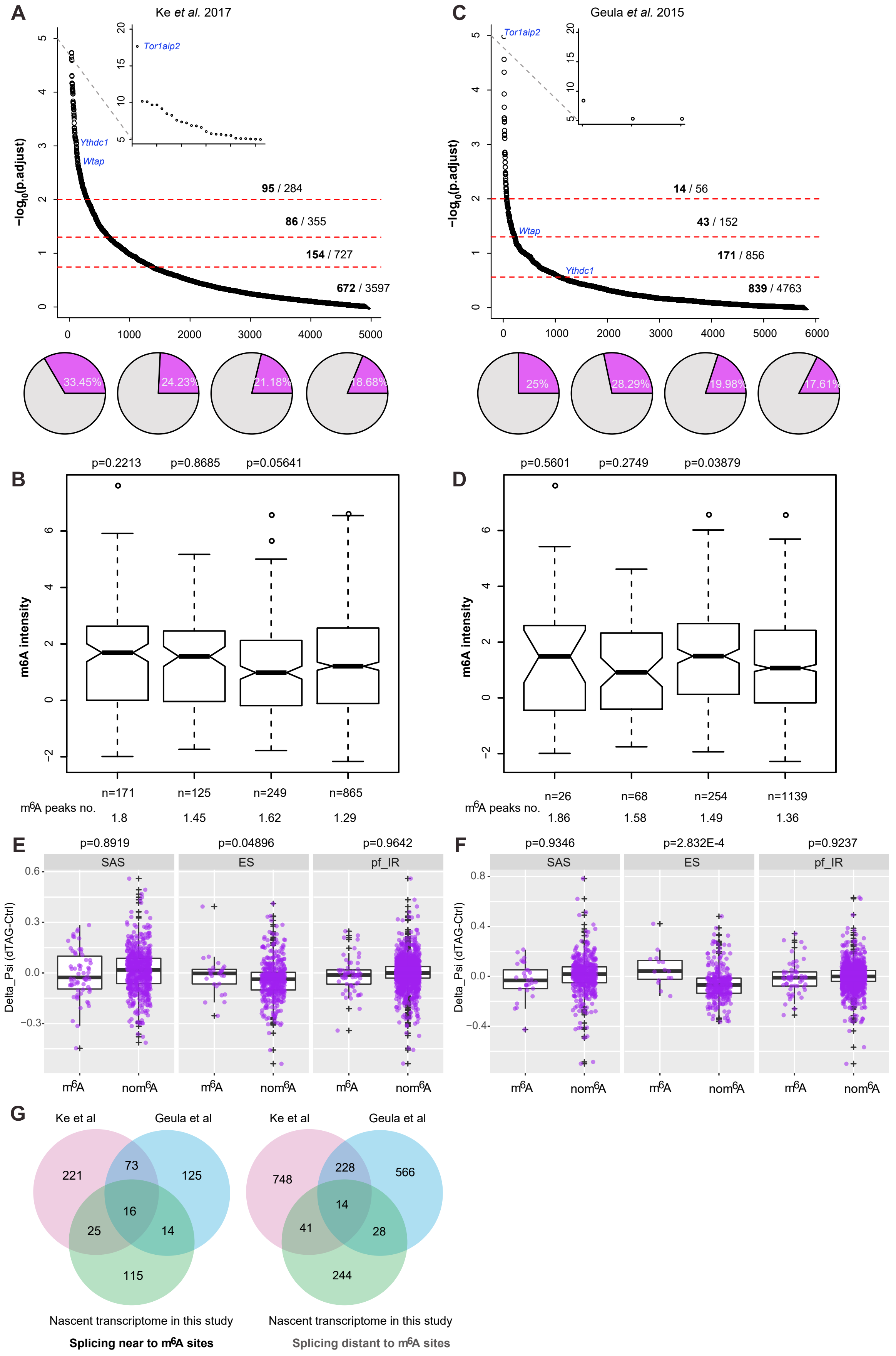

Supplemental Figure S11. Splicing changes in constitutive *Mettl3* KO mESCs, related to Figure 5 and 6.

(A-D) Same as Fig. 5B,C but using data from Ke et al. 2017, (A,B) and Geula et al. 2015, (C,D) generated from WT and *Mettl3* KO mESCs.

(E-F) Same as Fig. 5E but using data from Ke et al. 2017 (E) and Geula et al. 2015 (F).

(G) Venn diagrams showing the intersection of splicing clusters among the three datasets. The left shows splicing clusters have or neighbor m^6A sites, while right is for splicing cluster distant to m^6A sites.

Supplemental_Fig_S12

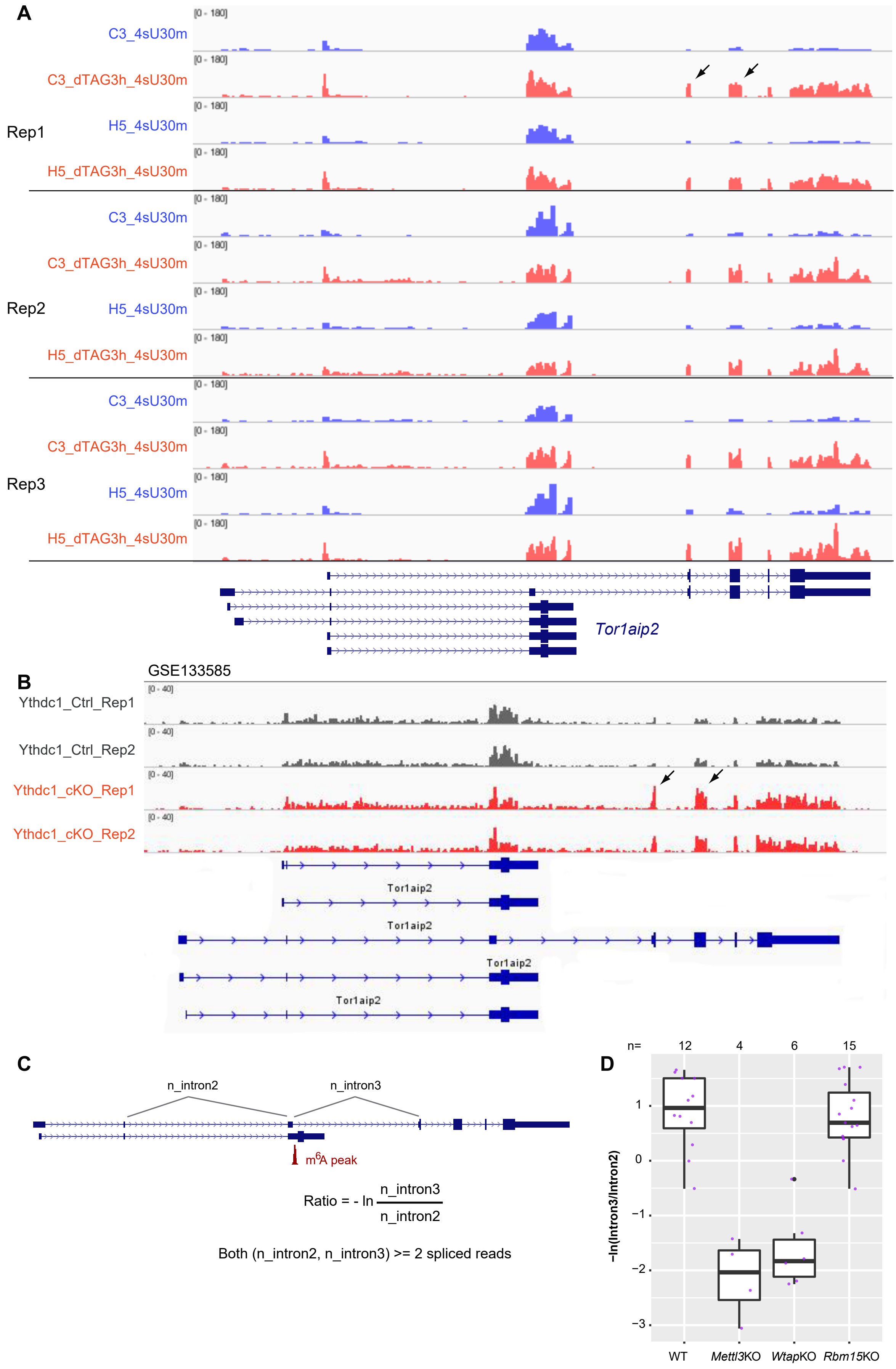

Supplemental Figure S12. Expression profile of *Tor1aip2* gene, related to Figure 6.

(A,B) IGV genome browser view of *Tor1aip2* gene expression profiles. Sample names are indicated on the left, gene annotation is shown below. Arrowheads indicate the induced long isoform. (A) Samples in three replicates with and without dTAG-13 treatment are shown in red and blue, respectively. Data are from 4sU-seq generated in this study. (B) Samples in two replicates with *Ythdc1* control and conditional knockout which are shown in grey and red, respectively. Data are nuclear RNA-seq from GSE133585 (Liu et al, 2020).

(C) Schematic about how to calculate the splicing ratio of intron 3 and 2 as -log(Intron3/Intron2,e).

(D) Boxplots showing the splicing ratio as (C). ChrRNA-seq datasets in which components of m^6A writer complex were perturbed (Nesterova et al. 2019). Samples were included only if at least 2 reads span each junction of intron2 and intron3.

Supplemental_Fig_S13

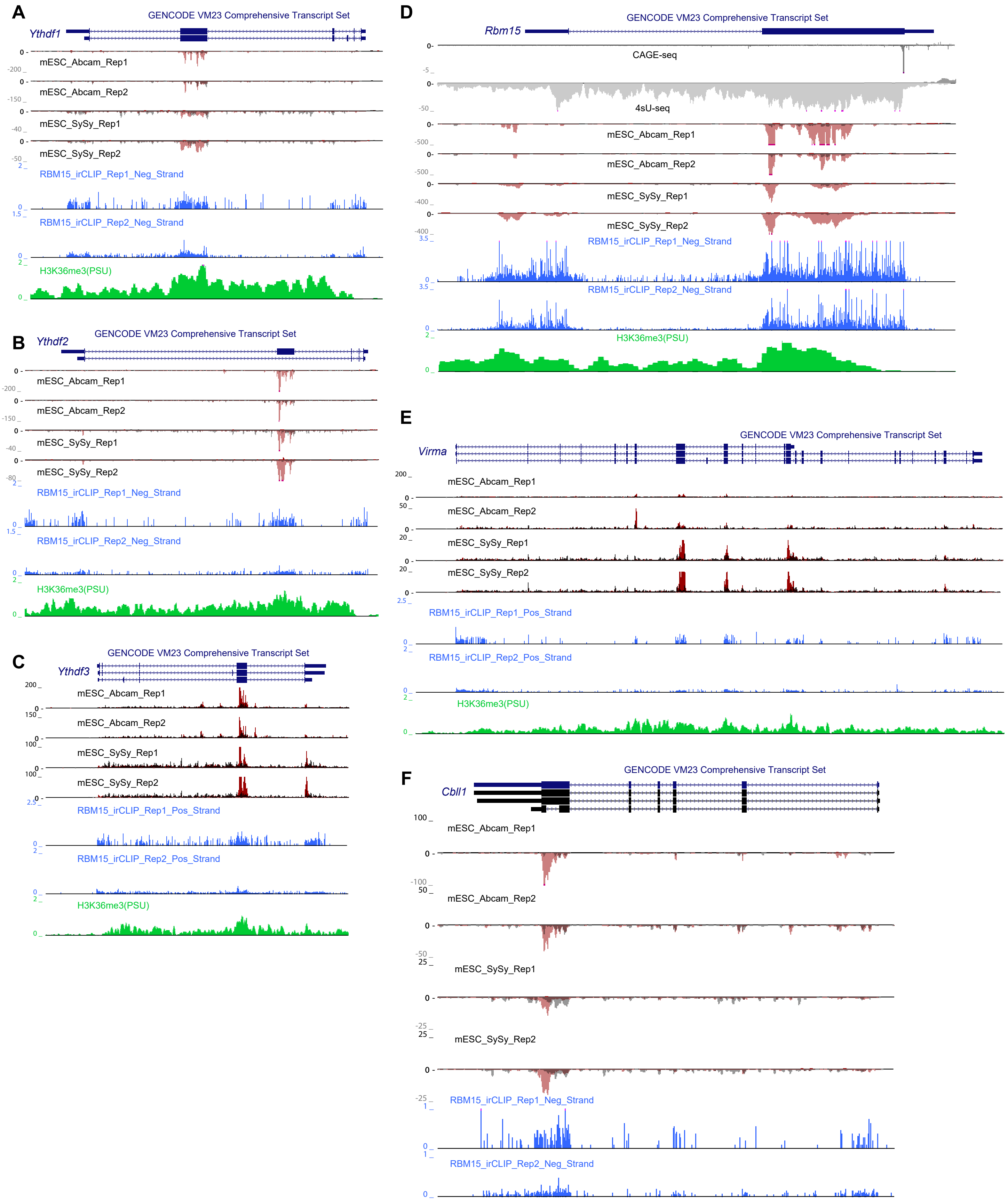

Supplemental Figure S13. Selected genes related to m^{6}A pathways are methylated, related to Figure 7.

(A-E) UCSC Genome Browser tracks for m^{6}A-related genes. (A-C) *Ythdf1/2/3*, (D) *Rbm15*, (E) *Virma,* (F) *Cbll1*. From top to bottom, they denote ChrMeRIP-seq (Abcam 2 replicates, SySy 2 replicates), RBM15 irCLIP-seq (2 replicates), and H3K36me3 ChIP-seq. In (D), the CAGE-seq (Wei et al. 2020) and 4sU-seq data are included.

Supplemental_Fig_S14

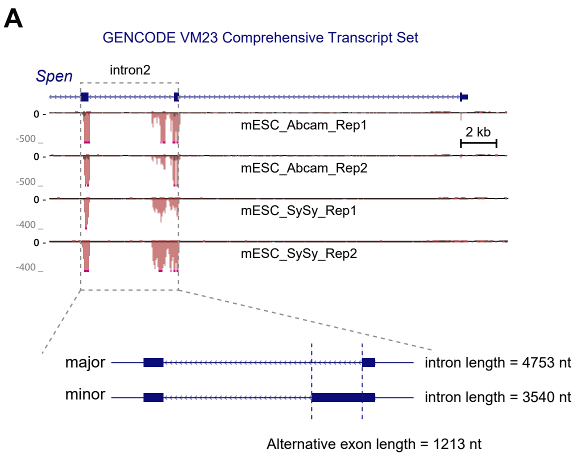

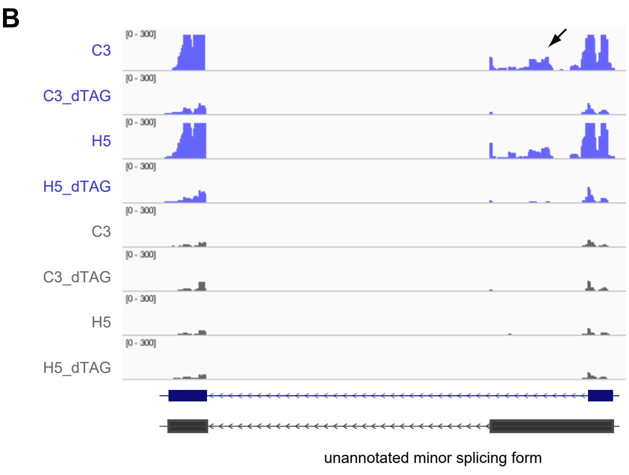

C

$$\text{Splicing choice} = \frac{\text{minor}}{\text{minor + major}}$$

METTL3 dTAG

	minor	major	Splicing choice		minor	major	Splicing choice
C3_Ctrl_1	30	38	44.12%	C3_dTAG_1	32	104	23.53%
C3_Ctrl_2	18	48	27.27%	C3_dTAG_2	31	193	13.84%
C3_Ctrl_3	64	153	29.49%	C3_dTAG_3	27	187	12.62%
H5_Ctrl_1	31	38	44.93%	H5_dTAG_1	29	77	27.36%
H5_Ctrl_2	56	131	29.95%	H5_dTAG_2	43	245	14.93%
H5_Ctrl_3	55	185	22.92%	H5_dTAG_3	19	255	6.93%

***Ythdc1* cKO**

	minor	major	Splicing choice		minor	major	Splicing choice
Control_1	7	15	31.82%	conditional KO_1	2	48	4.00%
Control_2	4	21	16.00%	conditional KO_2	2	32	5.88%

Supplemental Figure S14. m^{6}A mediates alternative 5'-splicing occurring at *Spen* intron2, related to Figure 6 and 7.

(A) ChrMeRIP-seq shows that *Spen* intron2 m^{6}A methylation as in Fig. 1G. Bottom shows the unannotated minor splicing form observed from 4sU-seq.

(B) IGV tracks showing that *Spen* intron2 RNA m^{6}A methylation (arrowhead) is sensitive to acute depletion of METTL3. Samples are indicated by name on the left.

(C) Tables showing the splicing choice score for the minor splicing form of *Spen* intron2 from 4sU-seq datasets (this study) (top) and conditional *Ythdc1* knockout nuclear RNA-seq from GSE133585 (bottom) (Liu et al. 2020).

Supplemental_Fig_S15

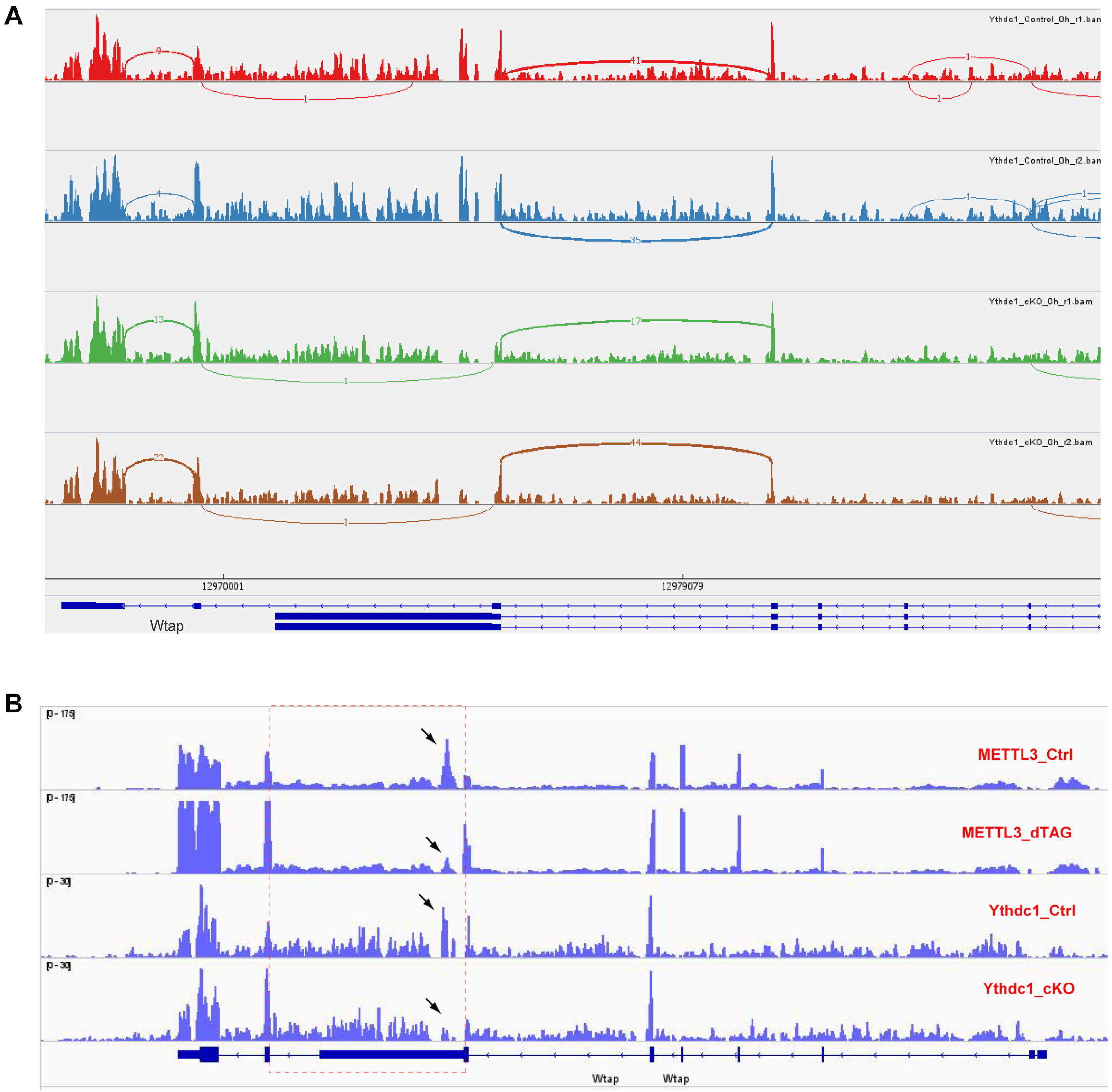

Supplemental Figure S15. m^{6}A mediates splicing changes at *Wtap* intron6, related to Figure 7.

(A) Sashimi plot showing the splicing of *Wtap* alternative last exon in *Ythdc1* control and conditional knockout of mESC nuclear RNA-seq (Liu et al. 2020). Top 2 tracks are 2 replicates of control samples while bottom 2 tracks are conditional knockout samples. The number of spliced reads is shown in the arcs. There are 3 main intron splicings as shown from left to right. *Wtap* is transcribed in an antisense manner. The left 2 splicings are increased upon loss of *Ythdc1*.

(B) Splicing of *Wtap* intron6 having m^{6}A modification. The pink dashed box indicates the intron having m^{6}A modification and arrowheads indicate the “partial” intron retention. The sample names are indicated by red. 4sU-seq for METTL3 dTAG study, and nuclear RNA for *Ythdc1* cKO study.

Supplemental Methods

Cell culture

All mouse embryonic stem cells (mESCs), except E14 mESCs, were grown in feeder-dependent conditions on gelatinized plates at 37°C in a 5% CO_2 incubator. Mitomycin C-inactivated mouse fibroblasts were used as feeders. mESCs medium consists of Dulbecco's Modified Eagle Medium (DMEM, ThermoFisher) supplemented with 10% foetal calf serum (Seralab), 2 mM L-glutamine (ThermoFisher), 1× non-essential amino acids (ThermoFisher), 50 µM *β*-mercaptoethanol (ThermoFisher), 50 g/mL penicillin/streptomycin (ThermoFisher), and 1 mL of Leukemia Inhibitory Factor (LIF)-conditioned medium made in-house. E14 cells were grown feeder-free in the same conditions and medium as above. *Xist* expression was induced by the addition of 1–1.5 µg/mL doxycycline (Dox) (Sigma-Aldrich, D9891) for 24 hours.

Molecular cloning and CRISPR-Cas9 mediated knockin

sgRNA targeting near *Mettl3* stop-codon was designed by online tool CRISPOR (Haeussler et al. 2016) (http://crispor.tefor.net/) and oligos were synthesised from Invitrogen, then cloned into pSpCas9(BB)-2A-Puro (PX459) V2.0 (Addgene #62988) backbone following instructions. A donor vector was built by Gibson assembly (NEB) of homology arms (~400 bp) PCR amplified from genomic DNA and the $FKBP12^{F36V}$ sequence amplified from plasmid pLEX_305-N-dTAG (Addgene # 91797) (Nabet et al. 2018). The Cas9-sgRNA-containing plasmid (Addgene #165420) and donor vector (Addgene #165421) were co-transfected at a molar ratio of 1:6 into XX mESCs cells on a 6-well plate using Lipofectamine 2000 according to the manufacturer's protocol (ThermoFisher). Transfected cells were passaged at different densities into three Petri dishes with feeders, 24h after transfection. The next day, cells were subjected to puromycin selection (3.5 µg/mL) for 48h and then grown in regular mESC medium until mESC colonies were ready to be picked and expanded. Western blot analysis was used to screen colonies for $FKBP12^{F36V}$ knock-in as this results in slower mobility of the METTL3_FKBP12^{F36V} protein compared to the wild-type protein in an SDS-PAGE gel and disappearance of the wild-type METTL3 band on western blot. Selected clones were further characterized by PCR of genomic DNA and Sanger sequencing to confirm correct knock-in and homozygosity. The sensitivity of selected METTL3_FKBP12^{F36V} clones to dTAG-13 treatment was validated by western blot. The plasmids used in this study are deposited in Addgene.

Western blot

Total cell lysates were resolved on a polyacrylamide gel and transferred onto PVDF or nitrocellulose membrane by semi-dry transfer (15V for ~50 minutes) or quick transfer. Membranes were blocked by incubating them for 1 hour at room temperature in 10 mL PBS, 0.1% Tween (TBST) with 5% w/v Marvell milk powder. Blots were incubated overnight at 4°C with the primary antibody, washed 4 times for 10 minutes with PBST and incubated for 1 hour with secondary

antibody conjugated to horseradish peroxidase. After washing 5 times for 5 min with PBST, bands were visualised using ECL (GE Healthcare). TBP serves as loading control.

Cell fractionation and Chromatin-associated RNA (ChrRNA) isolation

ChrRNA was extracted from a confluent 15 cm dish of mESCs. Briefly, cells were trypsinised and washed twice in cold PBS, and then lysed on ice in RLB buffer [10 mM Tris pH 7.5, 10 mM KCl, 1.5 mM $MgCl_2$, and 0.1% NP40] for 5 minutes, and nuclei were purified by centrifugation through a sucrose cushion (24% sucrose in RLB). The supernatant was kept as cytosolic fraction. The nuclei pellet was resuspended in NUN1 [20 mM Tris pH 7.5, 75 mM NaCl, 0.5 mM EDTA, 50% glycerol], then lysed with NUN2 [20 mM Hepes pH 7.9, 300 mM, 7.5 mM $MgCl_2$, 0.2 mM EDTA, 1 M Urea]. Samples were incubated for 15 minutes on ice with occasional vortexing, then centrifuged for 10 minutes at 2800g to isolate the insoluble chromatin fraction. The upper phase was kept as nucleoplasm fraction. The insoluble chromatin pellet was resuspended in TRIzol by passing multiple times through a 23-gauge needle. The disrupted chromatin pellet was either frozen in -80° C or freshly purified through standard TRIzol/chloroform extraction followed by isopropanol precipitation. Samples were then treated with two rounds of Turbo DNaseI (AM1907, Life Technology) in order to eliminate any potential DNA contamination. Concentration and quality of RNA isolated from the chromatin pellet were determined by Nanodrop and Agilent RNA Bioanalyzer.

4sU-RNA immunoprecipitation

4sU-RNA was generated and isolated essentially as described in (Rabani et al. 2011; Pintacuda et al. 2017). In detail, 4-thiouridine (4sU, Sigma-Aldrich, T4509) was dissolved in sterile PBS and stored at -20ºC. 4sU was thawed just before use and added to the cells in the growing media at a concentration of 500 µM. METTL3-FKBP12^{F36V} expressing cells (C3 and H5 clones) were treated with or without dTAG-13 (100 nM) for 3 hours, then exposed to 4sU-suplemented medium for 30 minutes. Cell culture medium was rapidly removed from cells and 5 mL of TRIzol reagent (Life Technologies) was added. Total RNA was isolated, and any potential DNA contamination was removed using Ambion DNA-free DNase Treatment kit (Life Technologies) according to the manufacturer's instructions. For each µg of total RNA, 2 µL of Biotin-HPDP (Pierce, 50 mg EZ-Link Biotin-HPDP), previously dissolved in DMF at a concentration of 1 mg/mL, and 1 µL of 10x Biotinylation buffer [100 mM Tris-HCl pH 7.4, 10 mM EDTA], was added. The reaction was incubated with rotation for 15 minutes at 25ºC. RNA was transferred to Phase Lock Gel Heavy Tubes (Eppendorf), and an equal volume of chloroform was added. After vigorously mixing, tubes were left incubating for 3 minutes at 25ºC and then centrifuged at 13,000 rpm for 5 minutes at 4ºC. The upper phase was transferred to new Phase Lock Gel Heavy Tubes, and chloroform added again. After further centrifugation, the upper phase was transferred to a tube containing an equal volume of isopropanol and 1/10 volume of 5 M NaCl. After inversion, the tubes were centrifuged at 13,000 rpm for 20 minutes at 4ºC. Supernatant was washed in 75% ethanol and resuspended in

water. Biotinylated 4sU-RNA was recovered using the μMacs Streptavidin Kit (Miltenyi), with a modified protocol. Per μg of recovered biotinylated 4sU-RNA, 0.5 μL of streptavidin beads were added, in a total volume of 200 μL. Samples were incubated with rotation for 15 minutes at 25ºC. μMacs columns supplied with the μMacs Streptavidin Kit were equilibrated in 1 mL of washing buffer [100 mM Tris-HCl pH 7.5, 10 mM EDTA, 1 M NaCl, 0.1% Tween 20] at 65ºC. Samples were added to the columns that were then washed 6 times with washing buffer, 3 times at 65ºC and 3 times at 25ºC. RNA was eluted in freshly prepared 100 mM DTT. RNA was further purified using the RNeasy MinElute Cleanup kit (Qiagen) according to the manufacturer's guidelines. 1 μL of 4sU-labelled RNA was quality-checked using the Agilent RNA 6000 Pico kit (Agilent technologies) according to the manufacturer's instructions and run on a 2100 Bioanalyzer Instrument (Agilent).

4sU-seq

Approximately 750 ng of 4sU-labelled RNA were subjected to sequencing library preparation by TruSeq Stranded total RNA LT Sample Prep with Ribo-Zero gold (Cat. No. RS-122-2301) according to manufacturer's instructions. Libraries were purified with AmPure XP beads and quantified by Qubit. Equal moles of libraries with different barcodes were pooled together based on the KAPA quantification (Roche) and sequenced using Illumina NextSeq500 (FC-404-2002) in paired-end mode (2×80).

emGFP-RBM15 irCLIP

The emGFP-PreScission-RBM15 cell line was described above. irCLIP were performed as (Zarnegar et al. 2016) with few modifications. Briefly, cells were grown with feeders and harvested after 24 hours Dox treatment. 10 million mESCs were washed with ice-cold PBS irradiated once with 150 mJ/cm^2 in a Stratalinked 2400 at UVC 254 nm on ice. Snap frozen pellets were stored at -80°C until use. Cell pellets were resuspended in 1 mL lysis buffer [50 mM Tris-HCl pH 7.4, 100 mM NaCl, 1% IGEPAL CA-630, 0.1% SDS, 0.5% sodium deoxycholate] supplemented with protease inhibitors, and transferred to a fresh 1.5 mL tube. Sonication was performed on a Bioruptor (Diagencode) for 10 cycles with alternating 30 secs on/ off at low intensity, and followed by RNase I (Thermo Scientific, EN0602) and Turbo DNase (Ambion, AM2238) treatment. Pre-washed antibody-conjugated beads were directly added to the RNase-treated lysate and rotated on a wheel overnight at 4ºC. After discarding the supernatant, the beads were washed 2× with high-salt wash buffer [50 mM Tris-HCl pH 7.4, 1 M NaCl, 1 mM EDTA, 1% IGEPAL CA-630, 0.1% SDS, 0.5% sodium deoxycholate]. 3'-end RNA dephosphorylation was performed on beads with PNK (NEB M0201L) for 20 min at 37ºC. Beads were further washed with 1× PNK wash buffer [20 mM Tris-HCl, pH 7.4, 10 mM $MgCl_2$, 0.2% Tween-20], 1× high-salt wash buffer, and 1× PNK wash buffer. Washed beads were subjected to RNA ligation with pre-adenylated infrared adaptor L3-IP-App (/5Phos/AG ATC GGA AGA GCG GTT CAG AAA AAA AAA AAA /iAzideN/AA AAA AAA AAA A/3Bio/, 1 μM) together with RNA ligase (M0204, NEB), and incubated overnight at 16°C in a thermomixer shaking at 1100 rpm. Beads were subsequently washed with 1× PNK

wash buffer, 1× high-salt wash buffer, 2× PNK wash buffer, and finally supplemented with 1× NuPAGE loading buffer. Supernatants were carefully collected and loaded on a 4-12% NuPAGE Bis-Tris gel (Invitrogen) according to the manufacturer's instructions. The gel was run at 180V until dye front reached the bottom. The protein-RNA-L3IR complexes from the gel were transferred to a Protan BA85 Nitrocellulose Membrane (Whatman) using the Novex wet transfer apparatus according to the manufacturer's instructions (Invitrogen; transfer for 2 hours at 30V). The entire lane above the expected molecular size of the protein of interest (emGFP-PreScission-RBM15) was cut from the membrane, and mixed with proteinase K (Roche, 03115828001). RNA was isolated by Phenol:Chloroform:Isoamyl Alcohol (Sigma-Aldrich P3803) using Phase Lock Gel Heavy tube (713-2536, VWR). The isolated RNAs were reverse transcribed by SuperScript IV with barcode primer (/5Phos/ WWW GTGGA NNNN AGATC GGAAG AGCGT CGTGAT /iSp18/ GGATCC /iSp18/ TACTG AACCG C). cDNA products were biotin captured with MyOne C1 SA-dynabeads, eluted, and subsequently circularised with CircLigase II (Epicentre). PCR amplification was obtained after 18-21 cycles. The amplified irCLIP libraries were purified using AmPure XP beads (Beckman Coulter) and subjected to size selection from TBE gel by cutting a 150-500 nt smear band. Library concentrations were quantified by Qubit and KAPA quantification kit (Roche), quality-checked by DNA Bioanalyzer (Agilent), and sequenced on an Illumina NextSeq500 machine using 75 bp single end sequencing.

Intronic patterns of m^{6}A methylation

Classification of peak summits (single nucleotides) were intersected with the selected MaxORF_LongestNcRNA GENCODE vM24 isoform (*intersectBed -a summits.bed -b *.GeneBed -s -wo*). Constitutive or alternative intron location was determined by whether the peak summit locates in intron for all the annotated comprehensive GENCODE vM24 isoforms or subsets of the isoforms respectively. PhastCons scores for multiple alignments of 59 vertebrate genomes to the mouse genome (phastCon60way) from UCSC were used (Pollard et al. 2010). For calculating conservation and GC contents, control region for this intronic m^{6}A methylation was chosen by randomly selecting the size-matched region from the same intron. For the relative intron length and position, random intron from the same transcript was chosen. Plots were generated by R package ggplot2. Scripts used for this analysis were attached.

Calibrated MeRIP-seq analysis

The raw reads from m^{6}A immunoprecipitation were mapped to mouse (mm10) and *Drosophila* (dm6) concatenated genome by STAR (v2.5.2b) (Dobin et al. 2013). The resulted alignment files (BAM) were then split into mm10 and dm6 alignment files according to the chromosome name. The read counts mapping to the dm6 were counted (Li et al. 2009) and used for subsequent normalization (Supplemental Table S1). The raw reads from input samples were mapped to the mm10 genome by STAR (v2.5.2b) (Dobin et al. 2013). Alignment files for m^{6}A IP and input

samples were also normalized in a conventional manner by 10 million mapped reads. The strand-specific bigwig files were generated and visualized by IGV (Robinson et al. 2011).

irCLIP-seq analysis

The irCLIP adaptors (sequence) from single-end sequencing reads were first cut by cutadapt (v1.12) (Martin 2011), and then collapsed by CTK toolkit to remove PCR duplication (Shah et al. 2017). The barcodes with UMI-containing 12 letters were moved to FASTQ header lines. The resulted single-end fastq reads, longer than 15 nt, were mapped to the mm10 genome by STAR (v2.5.2b) (Dobin et al. 2013) with key parameters (*--outSAMattributes All --outFilterMultimapNmax 1 --outFilterMismatchNmax 2 --alignEndsType EndToEnd --seedSearchStartLmax 15 --outWigType bedGraph read1_5p*). This setting is because RT stops were generated by reverse transcriptase around the UV crosslinked sites or crosslinking induced truncation sties (CITS), and RT-stops were main output when analysing the irCLIP-seq reads. The single nucleotide was considered as a CITS if there are no less than 3 stops (>=3) from independent strand-specific reads. In addition, crosslinking induced mutation sites (CIMS), particularly (deletion site) were also calculated. The single nucleotide was considered as a CIMS if this position has more than 2 deletions among total covered reads and the mutation rate is [0.01, 0.5], in order to avoid the potential contamination from the indels. Given that most of the RNA-binding proteins has 5-mer consensus motif, called CITSs or CIMSs from biological replicate 1 were kept if they were also called and within 5 nucleotides distance in biological replicate 2 (*closestBed -a CITS_Rep1.bed -b CITS_Rep2.bed -s -d | awk '$13<=5'*). The distribution of CITS and CIMS were analysed by the RNAmpp pipeline, described as above. Motifs around these CITSs or CIMSs were searched by Homer with key parameter (*findMotifsGenome.pl CITS.bed mm10 MotifOutput -rna*).

RBM15 binding, m^{6}A peak, and H3K36me3 modification

RBM15 CITS (>=3) called in 2 biological replicates were intersected with m^{6}A peaks from different confidence groups. Due to the strand-specificity, the closest distance between RBM15 binding and m^{6}A peak will be minus if RBM15 CITS located upstream of m^{6}A peak (*closestBed -a RBM15_CITS.bed -b m6A_peaks.bed -s -D a*) (Quinlan and Hall 2010). Strong RBM15 binding group was defined by no more than 1000 nt distance between RBM15 binding and m^{6}A peak. The density of RBM15 binding sites (CTIS>=3) 1000 bp flanking exonic m^{6}A peaks, intronic m^{6}A peaks, and intergenic m^{6}A peaks were plotted. BigWig files for H3K36me3 ChIP-seq data retrieved from ENCODE was generated in 10 bp bin size. The metagene profile and heatmap for RBM15 binding group and other groups were generated by deepTools (v3.4.3) (Ramirez et al. 2016). The matrix generating from negative strand bigWig files are combined with the one from positive strand, and with a brief modification to average two replicates, then subjected to plotHeatmap tool (Ramirez et al. 2016).

Differentially expressed genes analysis

4sU-seq were mapped to the mm10 genome by STAR (v2.5.2b) (Dobin et al. 2013) allowing multiple mapping (*--outFilterMultimapNmax 100 --outFilterMismatchNmax 5 --alignEndsType EndToEnd --winAnchorMultimapNmax 100*). TEtranscripts (Jin et al. 2015) was used to detect the differentially expressed genes and repeat elements.

Supplemental Table S1: Summary of ChrMeRIP-seq data, related to Figure 1.

Samples	total reads	mapped to mm10	Normalization Factor
SySy_IP_Rep1	171413985	75224035	7.5224035
SySy_IP_Rep2	167773086	78103747	7.8103747
SySy_input_Rep1	72543991	42643208	4.2643208
SySy_input_Rep2	69979632	42790747	4.2790747
Abcam_IP_Rep1	35247161	17359698	1.7359698
Abcam_IP_Rep2	26431065	12922923	1.2922923
Abcam_input_Rep1	18476488	12452285	1.2452285
Abcam_input_Rep2	20645009	15864869	1.5864869

Supplemental Table S2, Sequencing depth for normalization, related to Figure 4.

Samples	mm10	dm6	ScalingFactor (Calibrate)	ScalingFactor (Convention)
H5_input	8015137	NA	NA	0.8015137
H5_dTAG_input	9702459	NA	NA	0.9702459
C3_input	10305708	NA	NA	1.0305708
C3_dTAG_input	10439183	NA	NA	1.0439183
H5_m6A	32373084	2202971	11.01485	3.2373084
H5_dTAG_m6A	23644313	1180777	5.90388	2.3644313
C3_m6A	29186935	1133319	5.66659	2.9186935
C3_dTAG_m6A	38241256	1364155	6.82077	3.8241256

Supplemental Table S3: Primers used in this study to clone METLL3_FKBP12^{F36V} and PCR validation.

Primers used for sequencing the plasmid
>PQE30forward
CCCGAAAAGTGCCACCTG
>TNK78
GTTCAGGGGGAGGTGTGGGAGGTT

Primers used for sgRNA cloning
>Mettl3C_sgRNA_F
caccgTCTGAACGCTTAGCTTTGTA
>Mettl3C_sgRNA_R
aaacTACAAAGCTAAGCGTTCAGAc

Primers used for PCR cloning genomic regions near to Mettl3 stop codon, and also check the insertion
>Mettl3_CF
AATTGACGTGGACTGGGCAT
>Mettl3_CR
ATTGGGCTAGAGGGGAAACGA

Primers used for Gibson Assembly
>Mettl3C_Frag1F
TCCGCGCACATTTCCCCGAAAAGTGCCACCTGACGTACGTGGACTGGGCATAGTAGAG
>Mettl3C_Frag1R
catGCCTCCACTTCCACCTAAATTCTTAGGTTTAGAGATGATGCCGTCCG
>Mettl3C_Frag2F
TCTAAACCTAAGAATTTAGGTGGAAGTGGAGGCatgg
>Mettl3C_Frag2R
TTGTAACTAAGTGCGTCTAttccagttttagaagctccacatcg
>Mettl3C_Frag3F
gagcttctaaaactggaaTAGACGCACTTAGTTACAAAGCTA
>Mettl3C_Frag3R
GATGCGGCCGCGCTAGCACGCGTCAGCTGACTAGAGTACAGACTAATAAACCAGCAAACGTACATAGAATAAGCA
>PAM2Mutation_F
ggaaTAGACGCACTTCCTTACAAAGCTAAGCTTTCAGAGAGATGGGCTACAGGCCA
>PAM2Mutation_R
TGGCCTGTAGCCCATCTCTCTGAAAGCTTAGCTTTGTAAGGAAGTGCGTCTAttcc

Supplemental Table S4: Antibodies used in this study.

Antibody	Company	Catlog
METTL3	Abcam	ab195352
METLL4	Sigma-Aldrich	HPA038002
RBM15	Proteintech	10587-1-AP
WTAP	Proteintech	10200-1-AP
YTHDC1	Sigma-Aldrich	HPA036462
TOX4	Invitrogen	PA5-41558
TBP	Abcam	ab51841
EZH2	Cell Signaling	mAb#5246
m6A_Sysy	Synaptic Systems	202 003
m6A_Abcam	Abcam	ab151230
Anti-rabbit IgG, HPR, Donkey, WB (1:2000)	Amersham	cat#NA934V
Anti-mouse IgG,HRP, Sheep, WB (1:2000)	Amersham	cat#NXA931

Supplemental Data

Supplemental Data S1: Confidence m^{6}A peaks characterized in this study.

All the ChrMeRIP m^{6}A peaks (narrow peaks and peak summits) categorized in this study, based on genomic location and confidence group, are listed.

Supplemental Data S2: Differentially expressed genes between control and dTAG-13 treatment.

The differentially expressed genes and transposon elements called in C3 and H5 clones, separate and comment lists, are listed.

Supplemental Data S3: Splicing changes calculated from LeafCutter.

Splicing clusters, called by LeafCutter with p.value, between 6 untreated and 6 dTAG-13 treated samples, are listed.

Supplemental References

Batista PJ, Molinie B, Wang J, Qu K, Zhang J, Li L, Bouley DM, Lujan E, Haddad B, Daneshvar K et al. 2014. m(6)A RNA modification controls cell fate transition in mammalian embryonic stem cells. *Cell Stem Cell* **15**: 707-719.

Dobin A, Davis CA, Schlesinger F, Drenkow J, Zaleski C, Jha S, Batut P, Chaisson M, Gingeras TR. 2013. STAR: ultrafast universal RNA-seq aligner. *Bioinformatics* **29**: 15-21.

Geula S, Moshitch-Moshkovitz S, Dominissini D, Mansour AA, Kol N, Salmon-Divon M, Hershkovitz V, Peer E, Mor N, Manor YS et al. 2015. m6A mRNA methylation facilitates resolution of naïve pluripotency toward differentiation. *Science* **347**: 1002-1006.

Haeussler M, Schonig K, Eckert H, Eschstruth A, Mianne J, Renaud JB, Schneider-Maunoury S, Shkumatava A, Teboul L, Kent J et al. 2016. Evaluation of off-target and on-target scoring algorithms and integration into the guide RNA selection tool CRISPOR. *Genome Biol* **17**: 148.

Jin Y, Tam OH, Paniagua E, Hammell M. 2015. TEtranscripts: a package for including transposable elements in differential expression analysis of RNA-seq datasets. *Bioinformatics* **31**: 3593-3599.

Ke S, Pandya-Jones A, Saito Y, Fak JJ, Vagbo CB, Geula S, Hanna JH, Black DL, Darnell JE, Jr., Darnell RB. 2017. m(6)A mRNA modifications are deposited in nascent pre-mRNA and are not required for splicing but do specify cytoplasmic turnover. *Genes Dev* **31**: 990-1006.

Li H, Handsaker B, Wysoker A, Fennell T, Ruan J, Homer N, Marth G, Abecasis G, Durbin R. 2009. The Sequence Alignment/Map format and SAMtools. *Bioinformatics* **25**: 2078-2079.

Liu J, Dou X, Chen C, Chen C, Liu C, Xu MM, Zhao S, Shen B, Gao Y, Han D et al. 2020. N (6)-methyladenosine of chromosome-associated regulatory RNA regulates chromatin state and transcription. *Science* **367**: 580-586.

Martin M. 2011. Cutadapt removes adapter sequences from high-throughput sequencing reads. *EMBnetjournal* **17**: 10-12.

Nabet B, Roberts JM, Buckley DL, Paulk J, Dastjerdi S, Yang A, Leggett AL, Erb MA, Lawlor MA, Souza A et al. 2018. The dTAG system for immediate and target-specific protein degradation. *Nat Chem Biol* **14**: 431-441.

Nesterova TB, Wei G, Coker H, Pintacuda G, Bowness JS, Zhang T, Almeida M, Bloechl B, Moindrot B, Carter EJ et al. 2019. Systematic allelic analysis defines the interplay of key pathways in X chromosome inactivation. *Nature communications* **10**: 3129.

Pintacuda G, Wei G, Roustan C, Kirmizitas BA, Solcan N, Cerase A, Castello A, Mohammed S, Moindrot B, Nesterova TB et al. 2017. hnRNPK Recruits PCGF3/5-PRC1 to the Xist RNA B-Repeat to Establish Polycomb-Mediated Chromosomal Silencing. *Mol Cell* **68**: 955-969 e910.

Pollard KS, Hubisz MJ, Rosenbloom KR, Siepel A. 2010. Detection of nonneutral substitution rates on mammalian phylogenies. *Genome Res* **20**: 110-121.

Quinlan AR, Hall IM. 2010. BEDTools: a flexible suite of utilities for comparing genomic features. *Bioinformatics* **26**: 841-842.

Rabani M, Levin JZ, Fan L, Adiconis X, Raychowdhury R, Garber M, Gnirke A, Nusbaum C, Hacohen N, Friedman N et al. 2011. Metabolic labeling of RNA uncovers principles of RNA production and degradation dynamics in mammalian cells. *Nature biotechnology* **29**: 436-442.

Ramirez F, Ryan DP, Gruning B, Bhardwaj V, Kilpert F, Richter AS, Heyne S, Dundar F, Manke T. 2016. deepTools2: a next generation web server for deep-sequencing data analysis. *Nucleic Acids Res* **44**: W160-165.

Robinson JT, Thorvaldsdottir H, Winckler W, Guttman M, Lander ES, Getz G, Mesirov JP. 2011. Integrative genomics viewer. *Nature biotechnology* **29**: 24-26.

Shah A, Qian Y, Weyn-Vanhentenryck SM, Zhang C. 2017. CLIP Tool Kit (CTK): a flexible and robust pipeline to analyze CLIP sequencing data. *Bioinformatics* **33**: 566-567.

Wei G, Brockdorff N, Zhang T. 2020. The PWWP2A Histone Deacetylase Complex Represses Intragenic Spurious Transcription Initiation in mESCs. *iScience* **23**: 101741.

Zarnegar BJ, Flynn RA, Shen Y, Do BT, Chang HY, Khavari PA. 2016. irCLIP platform for efficient characterization of protein-RNA interactions. *Nat Methods* **13**: 489-492.
